# Comparative genome analysis reveals broad phylogenetic and functional diversity within the order *Nitrospirales*

**DOI:** 10.1093/ismejo/wraf151

**Published:** 2025-07-22

**Authors:** Linnea F M Kop, Hanna Koch, Daan Speth, Claudia Lüke, Eva Spieck, Mike S M Jetten, Holger Daims, Sebastian Lücker

**Affiliations:** Department of Microbiology, Radboud Institute for Biological and Environmental Sciences, Radboud University, Heyendaalseweg 135, 6525 AJ, Nijmegen, the Netherlands; Division of Microbial Ecology, Centre for Microbiology and Environmental Systems Science, University of Vienna, Djerassiplatz 1, 1030 Vienna, Austria; Doctoral School in Microbiology and Environmental Science, University of Vienna, Djerassiplatz 1, 1030 Vienna, Austria; Department of Microbiology, Radboud Institute for Biological and Environmental Sciences, Radboud University, Heyendaalseweg 135, 6525 AJ, Nijmegen, the Netherlands; Bioresources Unit, Center for Health & Bioresources, AIT Austrian Institute of Technology GmbH, Konrad-Lorenz-Straße 24, 3430 Tulln an der Donau, Austria; Division of Microbial Ecology, Centre for Microbiology and Environmental Systems Science, University of Vienna, Djerassiplatz 1, 1030 Vienna, Austria; Department of Microbiology, Radboud Institute for Biological and Environmental Sciences, Radboud University, Heyendaalseweg 135, 6525 AJ, Nijmegen, the Netherlands; Department of Microbiology and Biotechnology, University of Hamburg, Ohnhorststr. 18, 22609 Hamburg, Germany; Department of Microbiology, Radboud Institute for Biological and Environmental Sciences, Radboud University, Heyendaalseweg 135, 6525 AJ, Nijmegen, the Netherlands; Division of Microbial Ecology, Centre for Microbiology and Environmental Systems Science, University of Vienna, Djerassiplatz 1, 1030 Vienna, Austria; The Comammox Research Platform, University of Vienna, Djerassiplatz 1, 1030 Vienna, Austria; Department of Microbiology, Radboud Institute for Biological and Environmental Sciences, Radboud University, Heyendaalseweg 135, 6525 AJ, Nijmegen, the Netherlands

**Keywords:** *Nitrospira*, *Nitrospirales*, nitrification, nitrite oxidation, comammox, phylogenomic analyses, comparative genomics, quorum sensing

## Abstract

Nitrification, a key process in the nitrogen cycle, involves the oxidation of ammonia to nitrite and nitrate by a diverse group of chemolithoautotrophic microorganisms. The order *Nitrospirales* (referred to in literature as the genus *Nitrospira*), which includes both nitrite-oxidizing and complete ammonia-oxidizing bacteria, plays a central role in this process. We sequenced the genomes of nine *Nitrospirales* members, incorporating genomes from previously unsequenced taxonomic *Nitrospirales* lineages. A comprehensive genomic analysis of these new *Nitrospirales* was conducted, which included an examination of their habitat distribution, phylogenetic diversity, and functional capabilities. This was complemented by the construction of and comparison to a database of 446 non-redundant, high-quality *Nitrospirales* genomes. Our phylogenomic analysis uncovered the presence of additional unclassified lineages and provided a comparison between genome-based and 16S rRNA gene-based taxonomies. Whereas some *Nitrospirales* lineages seem to exhibit habitat preferences, others are found across a wide variety of ecosystems, suggesting a broad niche spectrum. This capacity to adapt to different environmental conditions is also reflected in the high variability and modularity of the respiratory chain and nitrogen assimilation mechanisms. Additionally, we found evidence of quorum sensing systems in species beyond lineage II, implying a broader ecological role for this communication mechanism within the *Nitrospirales*. Finally, we identified a set of conserved genes unique to nitrite oxidoreductase-containing *Nitrospirales,* providing insights into the emergence of this functional group. In conclusion, our study emphasizes the adaptability of the various nitrifying classes of the order *Nitrospirales* to diverse environments and reveals the presence of new taxonomic lineages.

## Introduction

Nitrification is a key process in the biogeochemical nitrogen cycle. The sequential oxidation of ammonia to nitrite and nitrate is catalyzed by different groups of chemolithoautotrophic microorganisms, including ammonia-oxidizing bacteria (AOB) and archaea (AOA) that catalyze aerobic ammonia oxidation, and nitrite-oxidizing bacteria (NOB), which are capable of oxidation of nitrite to nitrate. In addition, complete ammonia-oxidizing (comammox) bacteria can perform both steps of nitrification [1, 2]. Nitrification is essential for nitrogen removal in engineered systems such as wastewater and drinking water treatment plants (WWTPs and DWTPs, respectively) to prevent eutrophication, ensure the production of safe drinking water, and hinder bacterial regrowth in drinking water distribution systems [3, 4]. In addition to biotechnological applications, nitrification is a key nitrogen cycling process in natural systems and a major driver of primary production in various habitats [5, 6].

The ability to autotrophically oxidize nitrite has been observed in several phyla, including the *Pseudomonadota*, *Chloroflexota*, *Nitrospinota*, and *Nitrospirota* [7]. Nitrite oxidizers belonging to the genus *Nitrospira* within the order *Nitrospirales* are the most diverse known group of NOB [8–10], and members of this genus have been identified as comammox bacteria [1, 2]. A previous study suggested that the taxonomic range of NOB described as members of the genus *Nitrospira* needs to be expanded to the order level based on genome-based taxonomy [11]. Therefore, we will refer to all nitrite-oxidizing *Nitrospira* as members of the order *Nitrospirales* rather than the genus *Nitrospira*. Nitrifying *Nitrospirales* have been enriched or isolated from a variety of environments, including marine and hypersaline habitats [11–15], soil [16], geothermal springs [9], and engineered systems such as aquaculture biofilters [2, 17–19], water pipes [1, 10, 20], and DWTPs and WWTPs [16, 21–24].

Phylogenetic analyses of their 16S rRNA genes have shown that the order *Nitrospirales* can be divided into at least seven lineages [8–10, 21]. A lineage was defined as a monophyletic group comprising 16S rRNA gene sequences with ≥94.9% within-group and < 94% between-group sequence identity [8]. Lineages I, II, and IV are the most studied groups, as the majority of available isolates and enrichment cultures, as well as metagenome-assembled genomes (MAGs), belong to these lineages (Table S1) [1, 2, 8, 11–16, 19, 20, 22, 23, 25]. In contrast, fewer isolates and genome sequences exist for members of lineages V, VI, and VII (Table S1) [9, 10, 16, 21]. Lineage III even has no cultured representative [26], and only 16S rRNA gene sequences associated with this lineage are available.

In this study, we explored the phylogenetic and functional diversity of the nitrifying members of the order *Nitrospirales*. To this end, we expanded the genomic understanding of *Nitrospirales* by sequencing nine additional enrichment or pure cultures, most of them originating from understudied natural habitats. We obtained seven complete genomes, considerably increasing the number of closed genomes for this NOB group. Three of these high-quality genomes are from members of lineages without previously available genome information, including the novel lineage VIII represented by the *Nitrospiraceae* culture Kam-Ns4a [9]. In addition, we constructed and analysed a comprehensive genome database from publicly available sources comprising 445 high-quality, dereplicated *Nitrospirales* genomes, mainly MAGs. By constructing a comprehensive phylogenomic tree, we provide a comparison between the two methods of taxonomic classification, namely genome-based (GTDB) and 16S rRNA gene-based (*Nitrospira* lineage classification), and identify additional unclassified lineages as observed in previous studies (e.g. [21, 27]). However, our analysis extends beyond phylogeny to examine habitat distribution, respiratory chain modularity, nitrogen assimilation strategies, and quorum sensing mechanisms. In particular, we provide the first evidence for quorum sensing systems beyond lineage II, suggesting a broader ecological role for this communication mechanism within the *Nitrospirales*. Lastly, we investigated the transition from non-nitrite-oxidizing to putatively nitrite-oxidizing *Nitrospirales* and identified a gene set beyond the NXR genes that are inherent to this functional group.

## Materials and methods

### DNA isolation

In total, we sequenced the genomes of nine *Nitrospirales* enrichments (Tables S1 and S2), which were cultivated as previously described [9, 10, 13, 14, 16]. The genomes were sequenced using MiSeq (Illumina) and MinION (Oxford Nanopore Technologies) sequencing and assembled using the hybrid assembler unicycler (v.0.4.4) [28], as described in the Supplementary Materials and Methods. The assembled genomes of *Ca*. N. calida, *Ca*. N. bockiana, *Nitrospira* sp. Ecomares 2.1, *Nitrospira* sp. LUA16, *Nitrospira* sp. M1, *Nitrospira* sp. Nam74, and *Nitrospira* sp. Nam80 were complete and circular. Hybrid assembly of the *Nitrospira* sp. Kam-Ns4a genome resulted in one contig that could not be circularized, and the genome of *Ca*. N. salsa remained in five contigs, indicating incomplete genomes. All nine *Nitrospirales* genome sequences are available at the European nucleotide archive under the project accession number PRJEB85292.

### Generation of a *Nitrospirales* genome dataset

All available genomes of the order *Nitrospirales* were downloaded from NCBI (August 2023, *n* = 800). In addition, 81 *Nitrospirales* genomes were added from the IMG GEM dataset [29]. GTDB representatives of the other orders within the class *Nitrospiria* were included in the dataset and are referred to as” other *Nitrospiria*” genomes (2–01-FULL-66-17, JACQBW01, JACQBZ01, JACQCE01, JACRHA01, and SBBL01; *n* = 24). Furthermore, the genomes of three novel *Nitrospirales* enrichments and one isolate were downloaded from NCBI [11], and the genomes of Ca. *Nitrospira alkalitolerans* KS and *Nitrospira marina* Nb-295 were downloaded from the MicroScope platform [30]. Finally, we included the *Nitrospira* genomes (n = 398) that are part of the species representative sets of two recent meta-analyses, one global scale (SPIRE: a Searchable, Planetary-scale mIcrobiome REsource) [31], and one focused on soil (soil metagenome-assembled genome bin (SMAG) catalogue) [32]. This dataset does not yet include the recently published Ca. Nitrosymbion coscinodermae genome [33], but does contain the highly similar MAG (GCA_011523385.1; 97.9% ANI) published earlier [34]. All genomes were classified using the Genome Taxonomy Database Toolkit (GTDB-Tk; v2.1.1) classification workflow (classify_wf) using the r220 reference database [35]. Genome completeness and redundancy were assessed using the CheckM (v1.0.11) lineage_wf workflow [36] and the CheckM2 (v1.0.1) predict workflow [37]. Non-redundant genomes were selected using dRep with an average nucleotide identity (ANI) cutoff ≥99%, the “average” clustering algorithm, and” ANImf” for secondary clustering (v2.4.2) [38]. To include all enriched and cultivated *Nitrospirales* species in the dataset, we replaced the MAG_79 (GCA_009594875.1) with the well-annotated genome of *Nitrospira defluvii* (GCA_000196815.1) in the final dataset. In addition, the more recently uploaded genome currently designated as the reference genome for *Nitrospira defluvii* on NCBI (*Nitrospira* sp. ZN2, GCF_905220995.1) is affiliated with a distinct branch in the phylogenomic tree and clearly represents a different species than *N. defluvii* (Fig. S1). A list of all genomes, including results from GTDB-Tk, CheckM, and dRep, is provided in Table S3. A total of 446 non-redundant *Nitrospiria* genomes were then combined with the nine newly sequenced genomes of *Nitrospirales* cultures (Table S4). The Kamchatka hot spring enrichment Ns4a (16S rRNA gene accession HM485590) previously described [9] was renamed to Kam-Ns4a due to the name similarity to the family NS-4.

### Genome annotation

The genomes were annotated using DRAM [39] for gene calling with prodigal [40] and annotation against the KOfam [41] and Pfam [42] databases (Table S5). The annotation of key genes was manually curated using blastp (2.13.0+) [43] searches of representative proteins (e-value ≤0.00001, bitscore ≥80, percent identity ≥30%, query cover ≥80%). From the manually curated annotations, protein complex and pathway completeness were calculated based on the presence of the minimum number of required genes according to the corresponding Kyoto Encyclopedia of Genes and Genomes (KEGG) modules. Putative [NiFe] hydrogenases were identified in the annotation based on Pfam accession PF00374 and classified using the HydDB [44]. Proteins involved in guanidine import and degradation were searched using blastp (2.13.0+) [43] with query sequences for amino acid/polyamine/organocation permeases (APC superfamily; CUQ66147.1, CUS36629.1, CUS37195.1, QPD06046.1, THJ13106.1, THJ25539.1, NOT23800.1), guanidine carboxylase (NOT23797.1), carboxyguanidine deiminase A (CgdA; NOT23799.1), carboxyguanidine deiminase B (CgdB; NOT23798.1), allophanate hydrolase (TKS59337.1), and guanidinase (CUQ66148.1, CUS36632.1, CUS37197.1, QPD06045.1, THJ13102.1, THJ25543.1). Blastp results were filtered by e-value (≤0.00001), bitscore (≥80), percent identity (≥30%), and query cover (≥80%). The guanidinase-containing genomes of the non-comammox species *Ca*. Manganitrophus noduliformans Mn-1 and *Nitrospira* sp. Nam74 were screened for guanidine riboswitches using the infernal (v.1.1.5) [45] functions cmbuild, cmcalibrate, and cmsearch with the Rfam seed alignments guanidine-I (RF00442), guanidine-II (RF01068), and guanidine-III (RF01763). The manually curated annotations of key genes are provided in Table S6.

### Whole genome phylogeny

To generate a whole genome phylogeny, the selected genomes were annotated using anvi’o v8 [46], with gene calling using prodigal (v2.6.3) [40]. About 71 conserved marker genes were extracted from all genomes with hmmer (v3.3.2) [47] using hidden Markov models (HMMs) integrated in anvi’o. Hits that were erroneously included in the marker gene sets were removed based on manual inspection of the datasets and filtering by the expected protein length ranges using seqkit (v2.6.1) [48]. The curated datasets were aligned with muscle (v5) [49] and concatenated using catfasta2phyml (v1.2.0; https://github.com/nylander/catfasta2phyml). The concatenated alignment was used to calculate a phylogenetic tree using IQtree (v.2.2.2.7) [50], with the best mixture model identified by ModelFinder [51], constrained to using the models Blosum62, Dayhoff, LG, JTT, WAG, LG4M, and LG4X. A total of 1000 ultrafast bootstrap replicates were generated using UFBoot2 [52]. Alignments and tree files for the phylogenetic tree can be found at https://doi.org/10.6084/m9.figshare.c.7705802.v2.

An additional phylogenetic tree based on the GTDB-Tk bacterial marker gene set was calculated using genes identified and aligned with the GTDB-Tk (v2.4.0) identify and align functions [35]. The tree was calculated using IQtree (v2.1.4-beta), including ModelFinder with 1000 ultrafast bootstrap replicates, with Q.plant+F + I + G4 identified as the best model [51].

### 16S rRNA gene phylogeny

For the 16S rRNA gene-based phylogenetic tree, reference sequences were extracted from the Silva RefNR database (r138.1, taxonomy: *Nitrospirales*, sequence length >1399 nucleotides (nt), sequence quality >90, pintail quality >90) [53] and the MiDAS 4.8.1 database [54]. The reference sequences were then combined with the 16S rRNA gene sequences from the *Nitrospirales* genomes extracted using DRAM [39] and aligned using the SINA aligner [55]. 16S rRNA gene sequences extracted from *Nitrospirales* genomes that did not cluster within the order *Nitrospirales* were removed from the dataset. Alignment positions with gaps in more than 5% of sequences were removed using trimAl (v1.4.rev22, −gt 0.95) [56]. IQ-Tree (v1.6.12), including ModelFinder with 1000 ultra-fast bootstrap replicates, was used to construct the phylogenetic tree [51, 57], with SYM + I + G4 identified as the best model. Sequences belonging to orders other than the *Nitrospirales* within the class *Nitrospiria* (“Other”) were used as outgroup.

### Within-lineage sequence identity calculations

The 16S rRNA gene sequence identities between sequences belonging to the same lineage were calculated using Sequences Identities And Similarities (http://imed.med.ucm.es/Tools/sias.html) using default settings and the same alignment used to calculate the 16S rRNA gene tree. Similarities of sequences with a length <1200 bases were disregarded. Average amino acid identities (AAI) were calculated using ezAAI (v1.2.3). 16S rRNA gene sequence identities and AAI values within each lineage were visualized in R (v4.2.2) [58] using the geom_boxplot function of the ggplot2 (v3.3.5) package [59].

### Functional gene phylogenetic analyses

We performed phylogenetic analyses for the ureases (UreC), cyanases (CynS), and nitrite oxidoreductases (NxrA) using manually curated sets of reference sequences along with the amino acid sequences extracted from the *Nitrospirales* genomes. Additionally, the diversity and phylogenetic affiliation of CydA sequences encoding quinol-oxidizing *bd*-type oxygen reductases from the *Nitrospirales* genomes were analysed by inserting them into a previously published reference dataset [60]. For each tree, sequences were aligned using muscle [61], and phylogenetic trees were calculated using IQ-tree [50, 57], with the best evolutionary models selected by ModelFinder [51]. Further details on the reference database construction and tree calculation, including programme versions, can be found in the Supplementary Methods.

All trees were visualized and annotated using iTol (v6) [62].

### Comparative genomics with non-nitrite-oxidizing *Nitrospirota*

To assess the genes gained during the transition of *Nitrospirales* to their nitrite-oxidizing physiology, we compiled a dataset focused on this node in the *Nitrospirota* phylogeny. To this end, we obtained all genomes belonging to the JACQCE01 (*n* = 10) and JACQBZ01 (*n* = 14) orders within the *Nitrospirota*, as well as all genomes belonging to the basal UBA2166 (*n* = 4) and NS-4 (*n* = 28) families within the *Nitrospirales* (56 genomes total) from the GlobDB, a global species-dereplicated genome database (release 220; https://globdb.org/). We used the anvi’o databases available from the GlobDB to perform a pangenomics analysis as well as generate a concatenated marker gene phylogeny as described above for these 56 genomes. For the pangenome, all proteins in the 56 genomes were aligned using DIAMOND (v2.1.8) [63] to determine amino acid similarities, and hits were filtered using a minbit score of 0.3 [64]. Markov chain clustering (MCL) [65] was used to identify clusters of similar amino acid sequences, called “gene clusters”, using an MCL inflation parameter of 1.2. Anvi’o gene clusters strongly enriched in either the UBA2166 or the NS-4 clade were selected using the anvi’o interactive interface and extracted using the “anvi-summarize” programme. The extracted gene clusters were then filtered to remove any gene cluster containing sequences encoded in a genome outside the target group (UBA2166 or NS-4), as well as any gene cluster that is only present in a single UBA2166 genome. To identify NS-4 specific genes conserved in nitrite-oxidizing *Nitrospirales*, we used the amino acid sequences in each NS-4 specific gene cluster as references in a DIAMOND (v2.1.8) [63] blastp search, with all proteins encoded in all nitrite-oxidizing *Nitrospirales* (*n* = 431) as query (one search per gene cluster). We then, for each best hit, calculated the ratios between the alignment score and the theoretical maximum score and selected an alignment score ratio of 0.3 to keep proteins as true homologs. Finally, we included all anvi’o gene clusters present in at least 300 of the 431 NXR-containing *Nitrospirales* genomes in this study to account for the absence of genes due to genome incompleteness. For the concatenated marker gene phylogeny of the 56 basal genomes, we extracted 71 marker genes with HMMs included in anvi’o as described in the “whole genome phylogeny” section above, with the modification that the sequences were aligned using muscle (v3.8.1551) [61] and the resulting alignments were concatenated directly using the “anvi-get-sequences-for-hmm-hits” programme. The phylogeny was then calculated using Fasttree 2 [66], as integrated in the “anvi-gen-phylogenomic-tree” programme.

Environmental detection of the UBA2166 family was retrieved from the Sandpiper database (v0.3.0) of SingleM profiles from 248 905 metagenomes (https://sandpiper.qut.edu.au/) [67].

## Results and discussion

### *Nitrospirales* phylogeny and habitat range

**Figure 1:**
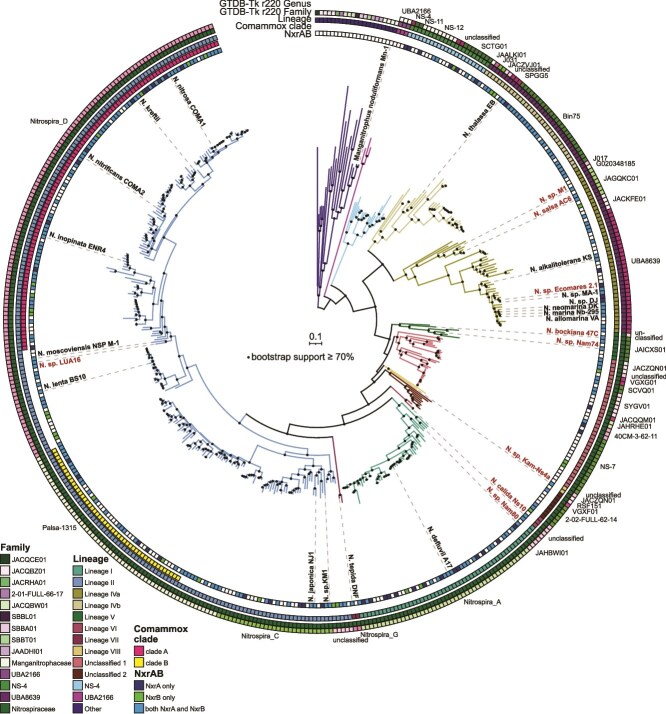
**Phylogenomic tree of non-redundant *Nitrospirales* genomes.** The tree includes genomes with ≥75% estimated completeness and ≤10% estimated redundancy and is based on the concatenated alignment of 71 core proteins. Black circles indicate bootstrap support ≥70% of 1000 ultrafast bootstrap replicates. The scale bar represents 10% sequence divergence. Tree branches are colored according to the lineage classifications. Newly sequenced genomes are labelled in red, and genomes of cultivated *Nitrospirales* are labeled in black. Labels of other *Nitrospirales* genomes and the prefix “*Candidatus*” were omitted for brevity; for details, see Fig. S1 and Table S1. Additional information on the presence of NxrAB subunits and comammox clade, lineage, and GTDB-Tk classifications (r220) is shown in the rings surrounding the tree. GTDB representatives belonging to orders other than the *Nitrospirales* within the class *Nitrospiria* are labeled as “other”, and their GTDB-Tk genus classifications were omitted for clarity.

To investigate the taxonomic and phylogenetic diversity within the order *Nitrospirales*, we constructed a database of non-redundant, publicly available *Nitrospirales* genomes (*n* = 446). In addition, we sequenced the genomes of nine *Nitrospirales* cultures, of which seven genomes are closed and two are nearly complete (one and five contigs, respectively; Table S1). These additional mostly complete genomes greatly expanded the dataset of reference genomes of cultured representatives from 18 to 27 (Table S1). Whereas the closed genomes cover only a fraction of the diversity of the order, in this study, we provide several genomes from lineages that were not previously sequenced (lineage V, *Ca.* Nitrospira bockiana and *Nitrospira* sp. Nam74; lineage VI, *Ca.* Nitrospira calida), including the genome of the enrichment culture *Nitrospira* sp. Kam-Ns4a within the putative lineage VIII (Fig. 1, Fig. S1) (Spieck et al., in preparation). Although in the study describing the enrichment culture *Nitrospira* sp. Nam74 was classified as lineage I [16], the genome-sequenced culture contained a lineage VI *Nitrospira* species, indicating a change in the dominant species within the enrichment culture. A comparison of the 16S rRNA gene-based and the phylogenomic trees shows that their overall structure is similar, with some exceptions (Fig. S2). Foremost, only phylogenomic analysis allows the reliable identification of comammox species within lineage II [3]. Whereas comammox organisms cluster closely together with canonical nitrite-oxidizing lineage II *Nitrospiraceae* in 16S rRNA-based analyses, they form two monophyletic clusters in the phylogenomic tree, corresponding to comammox clades A and B (Fig. 1, Fig. S1). According to GTDB taxonomy, clade A and clade B are classified as separate genera, both of which also harbor non-comammox *Nitrospira*.

Lineage V occupies a basal position relative to unclassified clusters 1 and 2 in the phylogenomic tree, whereas in the 16S rRNA gene tree, these unclassified clusters are positioned basal to lineage V. In addition, the sequences of unclassified cluster 1 do not form a monophyletic group in the 16S rRNA gene tree, in contrast to the phylogenomic tree (Fig. S2). Lineages I and VII cluster together in the 16S rRNA gene tree, with lineage II basal to them, whereas in the phylogenomic tree, lineages II and VII cluster together, with lineage I at their base. In a phylogenetic tree based on the marker gene set used by GTDB-Tk, lineages I and II cluster together with lineages VI and VII at their base, indicating that the evolutionary history of the underrepresented lineages remains ambiguous and awaits verification with additional genome sequences (Fig. S3). Evolutionary relationships within the middle portion of the trees, including lineages V, VI, and unclassified clusters 1 and 2, remain ambiguous, highlighting discrepancies between the 16S rRNA gene and phylogenomic approaches. These differences underscore the complexity of evolutionary inference and the potential limitations of single molecular markers in resolving phylogenetic relationships. Nevertheless, 16S rRNA gene phylogeny remains an important tool for the study of *Nitrospirales*, as lineage III is still represented solely by 16S rRNA gene sequences, and no genomes of this lineage are yet available. Lineage IV (family UBA8639) consistently emerges as the basal group to the *Nitrospiraceae* family in both trees. However, uncultured members of the family NS-4, which were recently identified as the most abundant NOB in floodplain sediment [68], also possess *nxrAB* genes (Fig. 1, Fig. S1), expanding the phylogenetic range of potentially nitrite-oxidizing organisms within the *Nitrospirales*.

There are significant differences when comparing the commonly used lineage and GTDB classifications. For example, genomes belonging to lineage II are distributed into four different genera according to GTDB. The available cultures of nitrite oxidizers currently affiliated with the “genus” *Nitrospira* are spread across ten different genera in two families within the order *Nitrospirales* (Fig. 1, Fig. S1). Whereas the current lineage classification is based on a threshold of >94.9% 16S rRNA gene sequence identity [8], several sequences are affiliated with lineages but show similarities below this threshold. This is particularly evident in the lineages I and IV (Fig. S4A, Table S7), indicating they should be split up into multiple lineages. In addition, the low average amino acid identities between genomes of the same lineage further exemplify their genomic diversity (Fig. S4B, Table S8), highlighting the need for a taxonomic reevaluation of this important nitrifying group, which should be a concerted effort of the nitrification research community.

**Figure 2:**
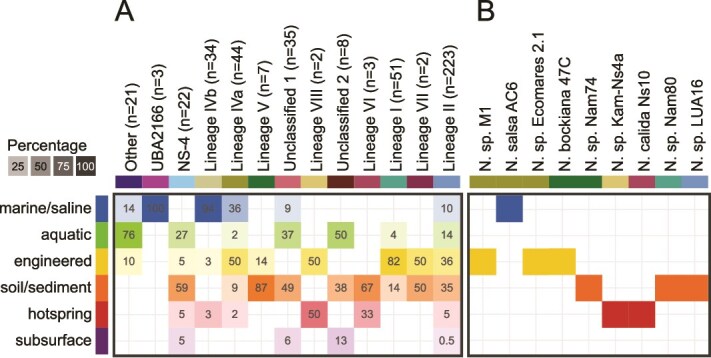
**Habitat distribution of *Nitrospirales* genomes. A)** Habitat distribution of the non-redundant *Nitrospirales* genomes (≥75% estimated completeness, ≤10% estimated redundancy). Values represent the percentage of genomes per habitat category for each lineage or cluster. Due to rounding, the values may not add up to exactly 100%. **B)** Habitats from which the newly sequenced *Nitrospirales* cultures were derived. The prefix “*Candidatus*” was omitted from the species names for brevity; for details, see Table S1. GTDB representatives belonging to orders other than the *Nitrospirales* within the class *Nitrospiria* are labelled as “other”.

The majority of GTDB-representative genomes belonging to the class *Nitrospiria* that are not from the order *Nitrospirales* (referred to in this article as the “other”) were recovered from aquatic systems (Fig. 2). All genomes belonging to the family UBA2166 within the order *Nitrospirales* were recovered from marine habitats. This family and the other *Nitrospiria* genomes do not encode nitrite-oxidizing proteins, representing non-nitrifying *Nitrospiria*. In contrast, as mentioned above the families NS-4 (lineage NS-4), UBA8639 (lineage IV), and *Nitrospiraceae* (lineages I, II, V, VI, VII, VIII, and unclassified clusters 1 and 2) possess the nitrite oxidation machinery and have been found in a wide variety of habitats, ranging from (freshwater) aquatic, marine, or saline habitats to hot springs, soil and sediment, subsurface, and engineered systems (Fig. 2). The majority of lineage I genomes have been recovered from metagenomes or cultures from engineered systems, mostly from WWTPs (Table S4). Our analyses show that there is a basal clade of lineage I genomes mostly found in soils, represented by our closed genome of *Nitrospira* sp. Nam80, which was enriched from a soil bio-crust sample [16]. Most lineage II genomes were obtained from engineered systems (35.87%) or soil samples (35.43%). Lineage IV can be divided into two groups (Fig. 1, Fig. S1) [69]. Both occur in marine or saline habitats (77.27% of lineage IVa and 97.06% of lineage IVb genomes), comprising natural as well as engineered systems. More than half of the lineage IVa genomes were obtained from saline engineered systems, including saline WWTPs, bioreactors, and biofilters (indicated as engineered systems in Fig. 2). Most lineage IVb genomes were associated with sponges (72.73%; Fig. 2, Table S4), although lineage IVb also contains free-living nitrite oxidizers [11]. The genomes from the mostly host-associated lineage IVb species are smaller than the lineage IVa genomes, corroborating the findings of Palomo and coworkers (Fig. S5) [27].

Lineage V and VI were mostly detected in soil samples, although this may not be representative due to the low number of genomes available for these lineages. The only representatives of lineage VII in our analyses, *Nitrospira tepida* DNF and the MAG SMAGOTU_20898, were retrieved from activated sludge and soil, respectively. Moreover, two additional lineage VII MAGs were recently obtained from a full-scale WWTP treating duck breeding waste [70], but were not yet included in our analyses. Lineage VIII comprises only two genomes, the hot spring-derived enrichment *Nitrospira* sp. Kam-Ns4a and a MAG derived from a WWTP (SPIREOTU_00841708). In addition to the described lineages, phylogenetic analyses revealed the presence of two additional groups whose taxonomy is still uncertain, as they lack cultured representatives (unclassified clusters 1 and 2). These were found primarily in natural samples such as freshwater, soil/sediment, and subsurface habitats. Whereas some lineages appear to have a habitat preference, such as lineage IV (marine/saline habitats) and lineage I (WWTPs and soil), most lineages have been found in various and diverse habitats, suggesting a broad niche spectrum of these *Nitrospirales*.

### Comparative genome analysis

#### Respiratory chain

**Figure 3:**
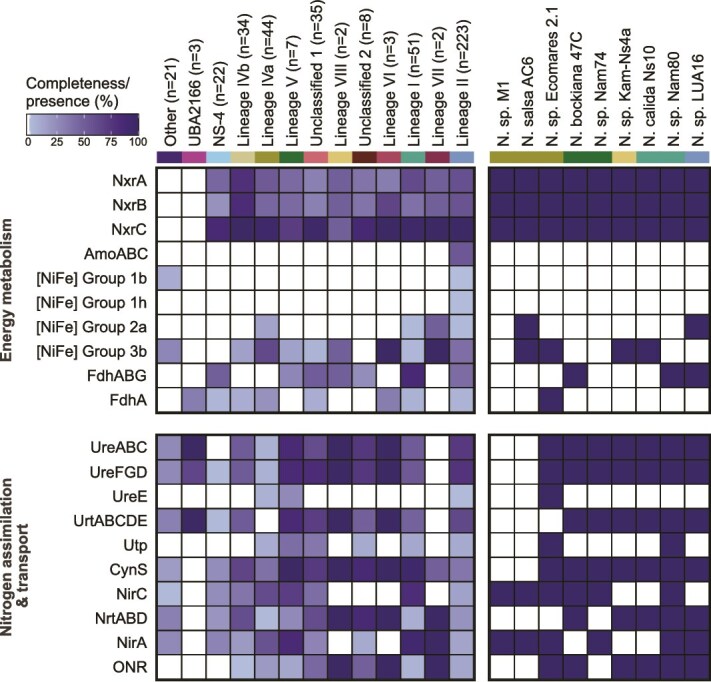
**Heatmap showing the presence of selected key genes involved in energy metabolism and nitrogen assimilation.** Complexes were counted as complete when ≥66% of genes were detected in a genome and counted as absent otherwise. White squares indicate the complete absence of the genes from a lineage or cluster. On the right, the presence or absence of genes in the newly sequenced *Nitrospirales* genomes is shown. The prefix “*Candidatus*” was omitted from the species names for brevity; for details, see Table S1. GTDB representatives belonging to orders other than the *Nitrospirales* within the class *Nitrospiria* are labeled as “other”. Other nitrogen metabolism genes are not included in this figure due to their low distribution. For detailed information on the genome annotations, see Tables S5 and S6. Abbreviations: NxrABC = nitrite oxidoreductase subunits A, B, and C; AmoABC = ammonia monooxygenase subunits A, B, and C; [NiFe] Group 1b, 1h, 2a, and 3b = [NiFe] hydrogenases belonging to the groups 1b, 1h, 2a, and 3b; FDH = canonical formate dehydrogenase; FdhA = *Nitrospira marina*-like putative formate dehydrogenase; UreABC = urease subunits A, B, and C; UreFGD = urease accessory proteins F, G, and D; UreE = urease accessory protein E; UrtABCDE = ATP-dependent ABC-type urea transporter; Utp = urea transporter (UT) family protein; CynS = cyanate lyase; NirC = nitrite transporter; NrtABD = ABC transporter, also called CynABD; NirA = ferredoxin-dependent nitrite reductase; ONR = octaheme cytochrome *c* nitrite reductase.

The predicted metabolic potential of the nine newly sequenced *Nitrospiraceae* genomes was compared with the annotations of non-redundant *Nitrospirales* genomes in our database. All of these genomes encode genes for aerobic respiration and CO_2_ fixation using the reverse tricarboxylic acid (rTCA) cycle (Table S6), which has been described in detail previously [71, 72]. As mentioned above, enzymes for nitrite oxidation are conserved in genomes of the families *Nitrospiraceae*, UBA8639 (lineage IV), and NS-4. The presence of multiple highly similar gene copies of the NXR [72] complicates the assembly and binning of the genes for this key enzyme, which explains the absence of single NXR subunits in many of the incomplete MAGs (Fig. 1, Fig. 3, Fig. S1). The topology of the NxrA tree differs significantly from the structure of the phylogenomic tree, indicating that NxrA is not a reliable taxonomic marker (Fig. S6). In this tree, several lineages, including lineages I and II, are split into multiple groups, and within lineage II, NxrA sequences from comammox clade A organisms are scattered across two distinct sequence clusters (Fig. S6). This discordance is further illustrated by the fact that whereas the NxrA copies of species such as *Nitrospira moscoviensis* (GCA_001273775) and *Nitrospira* sp. LUA16 show high similarity, other organisms, including *Ca*. Nitrospira calida, *Nitrospira tepida* (GCA_947241125), and *Nitrospira japonica* (GCA_900169565) possess several highly divergent NxrA copies affiliated with different clades of the tree. Here, it is tempting to speculate that these divergent NxrA paralogs may have different substrate affinities or preferences for the direction in which they operate [72, 73]. In conclusion, the evolutionary history of NxrA within the *Nitrospirales* is as puzzling as the potential origin of its transfer into the order [74]. In addition to nitrite, comammox organisms can also derive energy from the oxidation of ammonia using the ammonia monooxygenase (AMO) and hydroxylamine dehydrogenase (Hao) [1, 2]. As expected, AMO and Hao genes were only detected in genomes forming two monophyletic clusters within lineage II (Fig. S7), corresponding to comammox clades A and B (Fig. 1, Fig. S1).

The electrons from ammonia and/or nitrite oxidation are either transported directly to the terminal oxidase by the electron carrier cytochrome (cyt.) *c* or are used for NADH generation and CO_2_ fixation via reverse electron transport (see below). Electron transport requires several complexes of an apparently modular respiratory chain, although the modularity does not follow the phylogeny. For example, NADH dehydrogenase (NUO) is present in two or more copies in the majority of *Nitrospirales* genomes (Fig. S8). In addition to the canonical NUO with a fused NuoCD subunit, a second NUO complex encoding separate NuoC and NuoD, and two NuoM subunits (2 M) is hypothesized to be used for reverse electron transport to provide reduced ferredoxin required for CO_2_ fixation [71, 75]. Among the cultivated *Nitrospiraceae* and UBA8639 genomes, all encode this 2 M complex (Fig. S8). Furthermore, several genomes encode an additional NUO gene cluster containing fused NuoCD subunits. Lineage IV genomes and *Nitrospira* sp. Kam-Ns4a also encode additional putative *nuoL* and *nuoM* genes next to a multicomponent Na^+^:H^+^ antiporter (Mnh/Mrp), suggesting that they are additional *mnh*-like genes [12]. These transporters are mostly found in saline and alkaline environments, where they may play an important role in maintaining cell homeostasis.

Many AOB [76], anaerobic ammonium oxidizers [77], as well as the complete nitrifier *Ca.* N. kreftii [19] and the haloalkalitolerant *Ca*. N. alkalitolerans [12] possess a Na^+^-dependent NADH:quinone oxidoreductase (NQR) in addition to the canonical NUO complex I copies described above. The NQR is found in several lineage II and lineage IVa genomes (Fig. S9) and may represent an additional adaptation to saline and alkaline environments. Whether it is used during reverse electron transport for NADH production by consuming sodium motive force (SMF) or for exporting Na^+^ to generate a SMF for ATP production by the Na^+^-driven ATPase (see below) will have to be determined by further studies. More than half of the genomes encoding NQRs have been recovered from freshwater habitats or engineered systems, such as lakes, groundwater, WWTPs, or DWTPs, indicating that they are not a marine/saline-specific adaptation but may also confer a metabolic benefit in other habitats.

Additional modularity is found in complex III of the respiratory chain. In addition to the canonical *bc*_1_ complex, which is encoded in nearly all genomes, it was previously reported that *N. marina* and *Ca*. N. nitrificans encode a module of the structurally unrelated alternative complex III (ACIII) [78]. Similar complexes were identified in 47 of the genomes analysed, including *Ca*. N. bockiana, *Ca*. N. salsa, and the enrichment culture *Nitrospira* sp. Nam80 (Fig. S9). However, in all of these genomes, only genes similar to *actABCDE* were identified, with subunit B split into *actB1* and *actB2*, whereas the genes encoding the membrane subunits ActF and ActG were missing, as previously observed in other phyla [79] (Table S6).

In other organisms encoding an ACIII, an oxygen reductase is often found downstream of the *act* genes [80]. Similarly, almost all *Nitrospirales* genomes with an ACIII encode a cyt. *bd*-like oxygen reductase (CydAA’) of the OR-N5 type directly downstream (Table S6, Fig. S10). Whereas many CydA subunits are encoded in *Nitrospirales* genomes (Fig. S10), the NIDE0901-like OR-N2 subunit, together with an OR-N1-type CydA, is likely to form an OR-N-type CydAA’ terminal oxidase in the majority of *Nitrospirales* genomes [60, 71]. However, in addition to the OR-N5 cyt. *bd*-like oxygen reductases that were probably acquired together with the ACIII genes, almost half of the genomes encode an additional canonical CydAB cyt. *bd* oxygen reductase or a *cbb_3_* cyt. *c* oxidase (Fig. S9). All genomes of the 27 cultivated *Nitrospirales* species contain one of these additional oxygen reductases, with the exception of *N. lenta* BS10 (GCA_900403705), *Ca*. N. nitrosa COMA1 (GCA_001458735), and *Nitrospira* sp. Kam-Ns4a. This raises the question of whether the presence of multiple oxygen reductases is a selective advantage under standard cultivation conditions, resulting in a cultivation bias, or whether their absence in most MAGs is due to their incompleteness. Only 20.6% of lineage IVb genomes encode a *cbb_3_*-type cyt. *c* oxidase in addition to CydAA’ (OR-N cyt. *bd*-like oxidase), whereas more than 68% of lineage IVa and lineage I genomes have at least one additional (partial) oxygen reductase (Fig. S9). Both canonical cyt. *bd* and *cbb_3_*-type oxygen reductases may have high oxygen affinities [81–83], but large differences in affinities and regulation may exist [83]. The presence of several oxygen reductases with different oxygen affinities, in addition to the OR-N type CydAA’ terminal oxidase, whose oxygen affinity still remains unknown, may allow *Nitrospirales* bacteria greater flexibility in adapting to fluctuating environmental conditions. Whereas the *cbb_3_*-type cyt. *c* oxidase pumps protons into the periplasm and contributes to the proton motive force (pmf) required for ATP synthesis, the canonical cyt. *bd* oxidase contributes to the pmf only indirectly through transmembrane charge separation [84]. The canonical cyt. *bd* oxidases may also be involved in the detoxification of NO and CO as well as in oxidative stress defense, as shown for *E. coli* [85, 86].

In addition to oxygen, nitrate is an experimentally proven electron acceptor of *Nitrospira*. NXR is reversible and reduces nitrate, as observed for *N. moscoviensis* in the presence of hydrogen and formate [20, 72]. A recent genome-based study identified a cytoplasmic respiratory nitrate reductase (NarGHIJ) in a comammox MAG [87], but this metabolic feature is only present in a few *Nitrospirales* genomes (Fig. S7) and awaits further physiological analysis. The reduction of nitrate to nitrite might be coupled in some *Nitrospirales* with the dissimilatory reduction to ammonium in the presence of suitable electron donors. A few genomes (*n* = 23) contain *nrfAH* genes encoding a cyt. *c* nitrite reductase (Fig. S7), which would allow them to reduce nitrite produced by the NXR acting as a nitrate reductase in the presence of a suitable electron donor under anoxic conditions. The majority of these genomes belong to lineage II (*n* = 14), but *nrfAH* genes are also found in lineages I (*n* = 3), IVa (*n* = 1), VI (*n* = 2), VII (*n* = 1), the unclassified cluster 2 (*n* = 1), and a family NS-4 genome. Several of the closed genomes (*Nitrospira* sp. LUA16, *Ca*. N. calida, *N. tepida* (GCA_947241125, truncated *nrfA* gene [21]), and *N. inopinata* (GCA_001458695)) encode the *nrfAH* genes, confirming their presence in some *Nitrospiraceae* species and opening the possibility for future physiological studies of this intriguing metabolic feature. Although dissimilatory nitrite reduction generates ammonia, NrfAH may also be involved in nitrogen assimilation, as suggested for the comammox bacterium *N. inopinata* [1].

Variability of the respiratory modules is also found in the fifth and final complex of the respiratory chain, the ATP synthase (ATPase). In addition to the canonical F_1_F_o_-type ATPase encoded in almost all *Nitrospirales* genomes, 18% of the genomes encode an alternative N-type ATPase and 4.8% of the genomes encode a V/A-type H^+^-transporting ATPase with at least 50% completeness (Fig. S9). V/A-type ATPases can translocate Na^+^ or H^+^ across the membrane and can function bidirectionally [88, 89]. Thus, these additional N- or V/A-type ATPases may play a role in ATP synthesis and may also be used for ATP hydrolysis for pH homeostasis [90].

N-type ATPases were previously identified in the genomes of cultured *Nitrospirales,* potentially contributing to a SMF and salt resistance [11, 12, 78]. We found the MAG belonging to the *Nitrospiria* family JAADHI01 (GCA_945787655) and all lineage IV genomes to encode an N-type ATPase subunit c with a set of Na^+^-binding ligands. The majority of these genomes come from marine or saline habitats, suggesting that this complex may be used to pump out Na^+^ ions to increase salt tolerance, as has been shown for a similar ATPase [91]. In contrast, the majority of genomes with an N-type ATPase belonging to the family *Nitrospiraceae* lack the glutamate (E32, *Ilyobacter tartaricus* numbering), and the threonine (T670) in the Na^+^-binding motif EST—Y is replaced by a leucine in subunit c (Figs S9 and S11), similar to organisms with an F-type ATPase predicted to translocate H^+^ [92, 93], indicating that these may translocate H^+^ instead of Na^+^. These operons also lack the *atpR* gene for a transmembrane subunit, which is usually indicative of N-type ATPase operons. Still, the remaining genes (*atpGAFEBQCD*) are more similar to other N-type than to F-type ATPases, corroborated by the assignment of the *atpQ* genes (often annotated as *atpI*) to Pfam PF09527 [92]. Furthermore, these ATPases are found in genomes from different lineages and habitats, including DWTPs, soil, and rivers, leaving the evolutionary history and their adaptive advantage in different habitats unclear.

Finally, a small percentage of organisms encode a V/A-type ATPase, including the newly sequenced genomes of *Ca*. N. bockiana, *Ca*. N. calida, and *Nitrospira* sp. Kam-Ns4a (Fig. S9). Previously, these types of ATPases were found in the lineage VII organism *N. tepida* DNF [21] and the comammox MAG “RCA” [94]. The gene order in the lineage IV (family UBA8639) genomes differs from that in the remaining *Nitrospirales* genomes (Fig. S9). Together with the low sequence similarities between the ATPase subunits of these two lineages (e.g. for AtpA and AtpB, respectively, average sequence similarities of 51.48% and 55.51% between groups *vs.* 73.6%–74.8% and 78.7%–80.4% within groups with the same gene order), this suggests a different evolutionary history of this complex in the lineage IV genomes. Finding such differences underscores the importance of manual data curation, even in large-scale comparative genome studies. Although a recent study on the evolutionary history of ATPases concluded that the V/A-type ATPase evolved early in bacterial evolution and was lost in many bacterial lineages, there is evidence for horizontal gene transfer of this ATPase type in some lineages [95]. Thus, compared to other *Nitrospira*, lineage IV genomes may have acquired the V/A-type ATPase genes from a different donor, although further analysis is needed to confirm this. As it has been suggested that different evolutionary histories may reflect different modes of action [96], this would be an interesting avenue for future research into the functionality of V/A-type ATPases in the order *Nitrospirales*.

In summary, in addition to a core enzyme set for the canonical respiratory chain, *Nitrospirales* members possess several accessory modules, the distribution of which can vary either at the lineage or species level. This modularity allows flexibility in adapting to different environmental conditions. Different dehydrogenases and terminal oxidases have different proton translocation ratios [97], and the different types of oxygen reductases can be expressed under distinct conditions [98–101], allowing organisms to adapt to environmental fluctuations. Moreover, nitrate reduction under anoxic conditions in the presence of suitable electron donors like formate [72] makes *Nitrospirales* independent of their main substrates, oxygen and nitrite. In addition to oxidative phosphorylation, respiratory chain complexes may also have alternative roles such as reverse electron transport [12, 71, 102], pH homeostasis [12, 90, 91], or defense against oxidative or nitrosative stress [103, 104].

#### Alternative energy sources

*N. moscoviensis* uses a group 2a [NiFe] hydrogenase to oxidize hydrogen at atmospheric levels even in the presence of nitrite, using nitrate or oxygen as terminal electron acceptors [20, 105, 106]. Hydrogen oxidation likely enhances growth and survival under varying nitrite concentrations and provides additional energy that can be used for CO_2_ fixation and cell maintenance [106]. Group 2a [NiFe] hydrogenases were identified in only 13 *Nitrospirales* genomes, all but one of which were derived from enrichments or pure cultures (Fig. 3, Fig. S12).

In addition, one lineage II and two lineage IVa MAGs from environmental samples encode maturation proteases similar to the group 2a [NiFe] hydrogenase maturation protein in *N. moscoviensis*. This indicates that the remaining [NiFe] hydrogenase genes might be missing from these incomplete MAGs. The ability to oxidize hydrogen may confer an advantage under commonly used batch cultivation conditions, where nitrite is replenished only after its depletion, which may explain why this hydrogenase was found in several genomes from enrichments and pure cultures [12, 21, 105]. Other [NiFe] hydrogenases identified in *Nitrospirales* genomes belong to groups 1b, 1h, and 3b (Fig. 3). Group 1 [NiFe] hydrogenases were present in only three of the lineage II genomes (group 1h: GCA_002083365, SMAGOTU_00001; group 1b: SMAGOTU_12015) and two non-nitrifiers belonging to the family JACQBZ01 (other *Nitrospiria*; group 1b). These hydrogenase types have not been previously described in *Nitrospirales*, and their function remains to be elucidated. Similarly, group 3b [NiFe] hydrogenases, which are commonly found in the genomes of nitrifiers, have not been functionally characterized but have been hypothesized to play a role in the generation of NADH from H_2_ oxidation, H_2_ production, or reduction of elemental sulfur (S_0_) to H_2_S [12, 94, 107, 108]. Most genomes encoding group 3b [NiFe] hydrogenases belong to lineage II, specifically clade A comammox, and to lineage IVa (Fig. S12). Several genomes with a group 3b [NiFe] hydrogenase have only accessory proteins similar to those found in organisms with a group 2a [NiFe] hydrogenase, including group 2a [NiFe] hydrogenase maturation proteases, which are assumed to be hydrogenase-specific [109]. In addition, some genomes with both a group 2a and a group 3b [NiFe] hydrogenase encode only one hydrogenase maturation protease, and some with only a group 3b [NiFe] hydrogenase lack a maturation protease altogether. As this is also observed in high-quality closed genomes, this could also indicate that these group 3b [NiFe] hydrogenases employ an alternative maturation process or are no longer functional.

*Nitrospirales* have previously been shown to use pyruvate [8] or formate [110] as alternative carbon sources, and formate may also be used as an alternative energy source by some *Nitrospirales* populations [23, 72, 78, 110, 111]. Accordingly, NAD^+^-dependent formate dehydrogenases have been identified in many *Nitrospirales* genomes (Fig. S12). In addition, *N. marina* Nb-295, which utilizes formate as an energy and carbon source [78], encodes a divergent formate dehydrogenase, which was found in ~7.7% of the genomes presently analysed, many of them in lineage IVa. Despite the widespread occurrence of formate dehydrogenases in *Nitrospirales* genomes, this enzyme is rarely found in lineage IVb and comammox clade A genomes. The reason for this scarcity, however, is currently unclear.

About half of all genomes encode an Ech hydrogenase-related (Ehr) complex, which is similar to group 4 [NiFe] hydrogenases but lacks the [NiFe] binding sites [112] and was previously also referred to as a putative formate hydrogenlyase (Fig. S12) [71]. The function of this complex remains unknown, but due to the absence of [NiFe] ligands, a function as hydrogenase seems unlikely. The observation that Ehr gene expression was not increased in the presence of H_2_ in *N. moscoviensis* [105] supports this hypothesis.

#### Dissimilatory nitrogen metabolism

**Figure 4:**
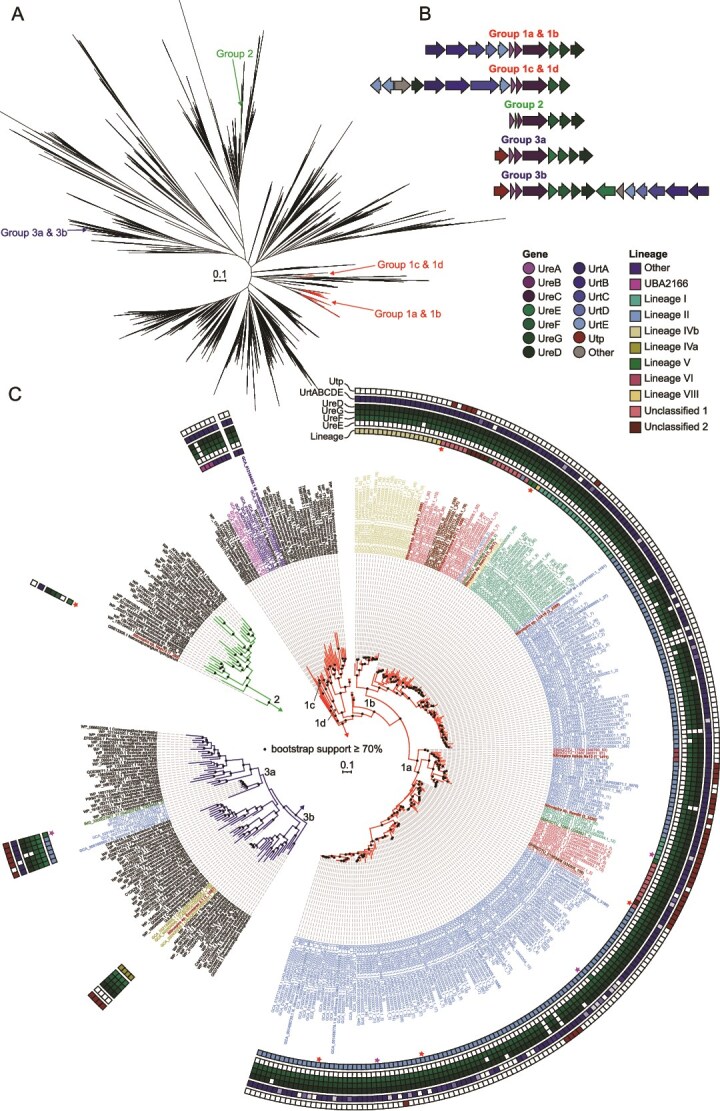
**Phylogeny and gene order of urease genes in *Nitrospirales* genomes. A)** Unrooted UreC tree showing the location of the *Nitrospirales* UreC sequences. The scale bar represents 10% sequence divergence. **B)** Schematic illustration of the most common urease and urea transporter gene orders in the different UreC groups. For detailed genome-level information, see Fig. S11. **C)** Sections of the UreC tree showing only the clusters in which *Nitrospirales* UreC sequences are found. Sequence labels are colored according to their lineage classification, with the exception of the newly sequenced genomes, which are shown in bold red text. Tree lines are colored according to the three groups shown in panel A. Black circles indicate bootstrap support ≥70% of 1000 ultrafast bootstrap replicates. The scale bar represents 10% sequence divergence. Additional information on the lineage and presence/absence of urease accessory genes and transporters is shown in the rings surrounding the tree. Red stars mark sequences of genomes that have multiple complete UreC copies. Purple stars mark genomes in which a second, partial *ureC* gene was found. GTDB representatives belonging to orders other than the *Nitrospirales* within the class *Nitrospiria* are labeled as “other”. Abbreviations: UreFGDE = urease accessory proteins; UrtABCDE = ATP-dependent ABC-type urea transporter; Utp = urea transporter (UT) family protein.

*Nitrospira* can degrade several organic N compounds, such as urea, cyanate, and guanidine, to produce ammonium [72, 113, 114], which can be either used for assimilation or, in the case of comammox organisms, as energy substrate to fuel nitrification. Urea hydrolysis is the most widespread capability for using an alternative ammonium source besides assimilatory nitrite reduction, as the majority of *Nitrospirales* genomes contain urease enzymes. However, genomes within the family NS-4 and most of lineage IVa (family UBA8639) lack urease (Fig. 3, Fig. S7). Phylogenetic analysis of the alpha subunit of urease (UreC) shows that *Nitrospirales* UreC are affiliated with several groups, mainly with groups 1a and 1b (Fig. 4A and C). These groups do not strictly correspond to phylogenetic relationships, and several genomes encode two copies of UreC affiliated with different groups. Lineages IVb and comammox clade B, along with related lineage II genomes, predominantly possess the ancestral group 1b UreC. Conversely, other lineage II genomes, including clade A comammox genomes, have a UreC belonging to group 1a. Sequences from these UreC groups are also found in other *Nitrospira* lineages, whereas UreC sequences from the family UBA2166 or other *Nitrospiria* orders fall into distinct branches (groups 1c and 1d; Fig. 4A and C). Furthermore, a few *Nitrospiraceae* UreC sequences are much more distantly related (groups 2 and 3; Fig. 4A and C). The complete genome of *Nitrospira* sp. Nam74 encodes a second copy of UreC that is unrelated to any of the other *Nitrospirales* sequences and occupies a basal position relative to a cluster of *Actinomycetes* sequences within group 2. In addition to the second UreC copy of the genome IMG_3300027907_21 (lineage V), UreC group 3 includes mainly sequences from *Nitrospiraceae* lineages II and IVa, like *Nitrospira japonica* NJ11, *Nitrospira* sp. M1, and *Nitrospira* sp. Ecomares 2.1 (Fig. 4). Lastly, some genomes contain a second partial *ureC* sequence, precluding their inclusion in phylogenetic analyses. In addition to UreC phylogeny, additional genes involved in urea metabolism suggest that *Nitrospiraceae* genomes possessing group 3 UreC enzymes acquired a urea metabolism gene cluster by horizontal gene transfer, originating from a source distinct from the majority of urease-positive *Nitrospirales*. Due to the cytoplasmic localization of urease, urea transporters are required, and we identified two different types in *Nitrospirales* genomes. The high-affinity ABC-type urea transporter (UrtABCDE) is employed by the majority of *Nitrospirales*, whereas some genomes encode a low-affinity transporter homologous to eukaryotic urea transporters (Utp) [115]. The transporter gene(s) form a gene cluster together with the urease (*ureABC*) and accessory genes. The ABC-type urea transporter is mostly located upstream of *ureABC*, except for group 3 *ureC*-containing gene clusters, where the *urtABCDE* genes are located downstream of the *ureABC* genes and the urease accessory proteins or on a separate contig (Fig. S13). Several genomes encoding a group 1a or 1b UreC also have an *utp* gene located between the *urtABCDE* and the *ureABC* gene clusters. Furthermore, the urease accessory proteins necessary for nickel (Ni^2+^) incorporation into the urease [116, 117] encoded by the genes *ureF*, *ureG*, and *ureD* are found in almost all genomes, whereas the accessory protein UreE is absent from most genomes except those encoding a group 3 UreC and one genome with a group 1b UreC (GCA_015904025.1; Fig. 3, Fig. S13, Supplementary Results and Discussion).

In addition to urea, cyanate can be used as an ammonium source. About half of the analysed *Nitrospirales* genomes encode cyanases (CynS) for the conversion of cyanate to ammonium and CO_2_ (Fig. 3, Fig. S7). It has been shown that nitrite-oxidizing *Nitrospira* can degrade external cyanate [113], possibly for detoxification or to obtain ammonium for nitrogen assimilation. In addition, recent studies have shown that also few comammox *Nitrospira* possess CynS [87, 118], which may allow them to use cyanate degradation to drive ammonia oxidation, as also observed in AOA [113]. Some marine AOA capable of cyanate degradation lack canonical CynS sequences, suggesting that alternative pathways for cyanate utilization may exist in other nitrifiers [119]. Phylogenetic analysis (Fig. S14) indicates the presence of two different types of cyanases within the phylum *Nitrospirota*. Most *Nitrospirales* CynS sequences cluster together, including the comammox CynS sequences, which, however, form their own branch within lineage I and distinct from other lineage II *Nitrospiraceae*. In addition, a small group of genomes, mainly belonging to lineage IVa and lineage V, encode CynS sequences that are more similar to *Nitrospina* proteins.

As cyanases are cytoplasmic enzymes, the bacteria rely on the transport of external cyanate into the cell. About half of the cyanase-encoding *Nitrospirales* encode an ABC transporter (CynABD/NrtABD) next to the *cynS* gene. The nitrite transporter (*nirC*) gene, located downstream of *cynS,* may be another candidate for cyanate import (Fig. 3, Fig. S7) [120]. Among the cyanase-encoding *Nitrospirales*, 9.5% of the genomes encode both CynABD and NirC next to CynS. However, most genomes have one of the two cyanate transporters, and there are clear differences between lineages: for instance, cyanase-encoding lineage I genomes mostly encode NirC (83.7% of cyanase-encoding lineage I genomes). In contrast, almost all lineage IVb genomes have an ABC transporter next to their *cynS* (91.3%). In almost all genomes with *Nitrospina*-like CynS sequences, the NirC is encoded elsewhere in the genome, including the complete genome of *Nitrospira* sp. Ecomares 2.1. As both the ABC transporter and NirC can also transport nitrate or nitrite [121, 122], the proteins encoded in other genomic locations may have a different function (Fig. S14). Thus, the capability for importing and degrading external cyanate needs to be confirmed experimentally in future studies for these organisms.

Recently, the comammox bacterium *N. inopinata* was found to grow on guanidine as its sole energy and nitrogen source [114]. Pathways for guanidine degradation were found in almost all comammox genomes, with the majority encoding an amino acid/polyamine/organocation (APC) permease and a guanidinase for guanidine import and degradation, as in *N. inopinata* (Table S6) [114]. In addition, three genomes of canonical nitrite oxidizers and *Ca*. Manganitrophus noduliformans Mn-1 (GCA_012184425) contain putative genes for guanidine degradation: the guanidinase in *Ca*. Manganitrophus noduliformans Mn-1 and *Nitrospira* sp. Nam74, and the deiminase B (CgdB) in GCA_020697375 and GCA_024998595 (both lineage I). The two hits to CgdB in GCA_020697375 and GCA_024998595 only have low identities to the query sequence (30.6%–31.4%), but high similarities to urea carboxylase-associated family proteins based on a BLASTp survey against the NCBI nr database. As no other guanidine degradation enzymes could be identified, these are likely false-positive hits. The guanidinase-like genes in *Ca*. Manganitrophus noduliformans Mn-1 and *Nitrospira* sp. Nam74 have higher sequence identities to the guanidinase of *N. inopinata* (54.3%–62.3% to CUS37197.1), but no genes involved in guanidine transport and no guanidine riboswitches could be identified in these genomes. Considering that the *Ca*. Manganitrophus noduliformans Mn-1 genome was derived from a binary co-culture [123] and the genome of *Nitrospira* sp. Nam74 is closed, misbinning is unlikely, and the role and functionality of these genes remain to be elucidated.

#### Assimilatory nitrite reduction

It was previously noted that genes for nitrogen anabolism are often found in a conserved gene cluster present in many canonical nitrifiers belonging to the lineages I, II, and VII, but not in comammox *Nitrospira* [21, 124]. We found a similar clustering pattern in several genomes belonging to other lineages (including lineage VI, NS-4, unclassified clusters 1 and 2) but not in lineage IV and V genomes (Table S6).

Besides the ferredoxin-dependent nitrite reductase NirA, an octaheme cyt. *c* nitrite reductase (ONR) may be used to facilitate nitrogen assimilation during growth on nitrite as the sole energy and nitrogen source [72]. Expression of *nirA* in *N. tepida* (lineage VII) and *onr* in *N. moscoviensis* and *N. japonica* NJ1 (lineage II) in the absence of ammonium supports their role in assimilatory nitrite reduction [21, 73, 125]. One of these two nitrite reductases is encoded in 41% of genomes, and only a small percentage (4%) have both genes, which may allow these organisms to adapt to fluctuating environmental conditions (Fig. 3) [21]. The majority of genomes with neither NirA nor ONR are lineage II comammox genomes. Most genomes with an ONR encode a Rieske-cyt. *b* complex (*petBC*) next to it, leading to the hypothesis that this complex may be the electron donor for nitrite reduction to ammonia by the ONR [72]. A quarter of the ONR-encoding genomes lack these *petBC* genes but instead encode a NapC protein next to the ONR. All of these genomes belong to lineage IV, and it is tempting to speculate that this cyt. *c*-type protein may serve as electron shuttle from the quinone pool to the ONR for nitrite reduction in these organisms.

#### Sulphur metabolism

Genes involved in sulphur metabolism have been previously identified in nitrifiers, including *Nitrospirales* genomes [11, 78, 108, 126, 127]. The presence of the putative periplasmic sulfite cyt. *c* oxidoreductase (SorAB), which may be involved in the oxidation of sulfite to sulfate but may also have a function in sulfur assimilation [108], is largely restricted to lineage IV genomes, with the exception of two genomes from other lineages (*sorA* in SPIREOTU_00061305 (family NS-4) and *sorAB* in SMAGOTU_12015 (lineage II), both on very short contigs). In lineage IVb, *sorAB* genes are found only in the genomes basal to the lineage and are absent in most genomes recovered from sponges (Table S6). In contrast, sulphide/quinone oxidoreductases (SQRs), which are involved in the oxidation of hydrogen sulphide to elemental sulphur or polysulphide, or sulfide detoxification, are abundant in lineage VI, lineage I, and part of lineage II genomes, but are found in only a few lineage IV genomes and are absent from almost all clade B comammox genomes. It will be interesting to see if the presence of SQR confers the ability to oxidize sulphide, as has been observed in the gammaproteobacterial nitrite oxidizer *Nitrococcus mobilis* Nb-231 [126].

#### Adaptation to marine environments

As the majority (85.9%) of lineage IV genomes originate from marine habitats, we analysed their mechanisms for coping with elevated salinity. The choline transporter (BetT) for the import of choline, the precursor of the osmoprotectant glycine betaine, and the multicomponent Na^+^/H^+^ antiporter (MnhBCDEFG) are found almost exclusively in lineage IV genomes, plus a small number of lineage II genomes, and some genomes belonging to the most basal group within the unclassified cluster 1. In addition, *Ca*. N. bockiana and *Nitrospira* sp. Kam-Ns4a (lineages V and VIII) encode MnhBCDEFG (Fig. S15). Strikingly, almost all genomes recovered from sponges in lineage IVb lack these transport systems, suggesting that they may rely on other osmoregulation mechanisms. The osmoprotectant uptake transport system OpuABCD is more widespread in other lineages but is also conserved in nearly all lineage IV genomes (Fig. S15). Similarly, the biosynthesis pathway for the osmoprotectant trehalose is conserved in most *Nitrospirales* genomes.

#### Quorum sensing

Quorum sensing is a common communication and gene regulation mechanism in bacteria that controls various cell density-dependent functions, such as biofilm formation, stress adaptation, as well as virulence factor production and bioluminescence [128]. Generally, quorum sensing systems include the production of an autoinducing signal molecule such as acylated homoserine lactone (e.g. by LuxI) and a transcriptional regulator (e.g. LuxR) responsible for orchestrating specific cellular responses [129]. It was previously shown that quorum sensing can affect nitrogen metabolism in the nitrifier *Nitrobacter winogradskyi* [130]. In the *Nitrospirales*, N-acyl-L-homoserine lactone (AHL) synthases and AHL production have only been described in the lineage II members *N. moscoviensis* and *N. japonica* [125, 128].

**Figure 5:**
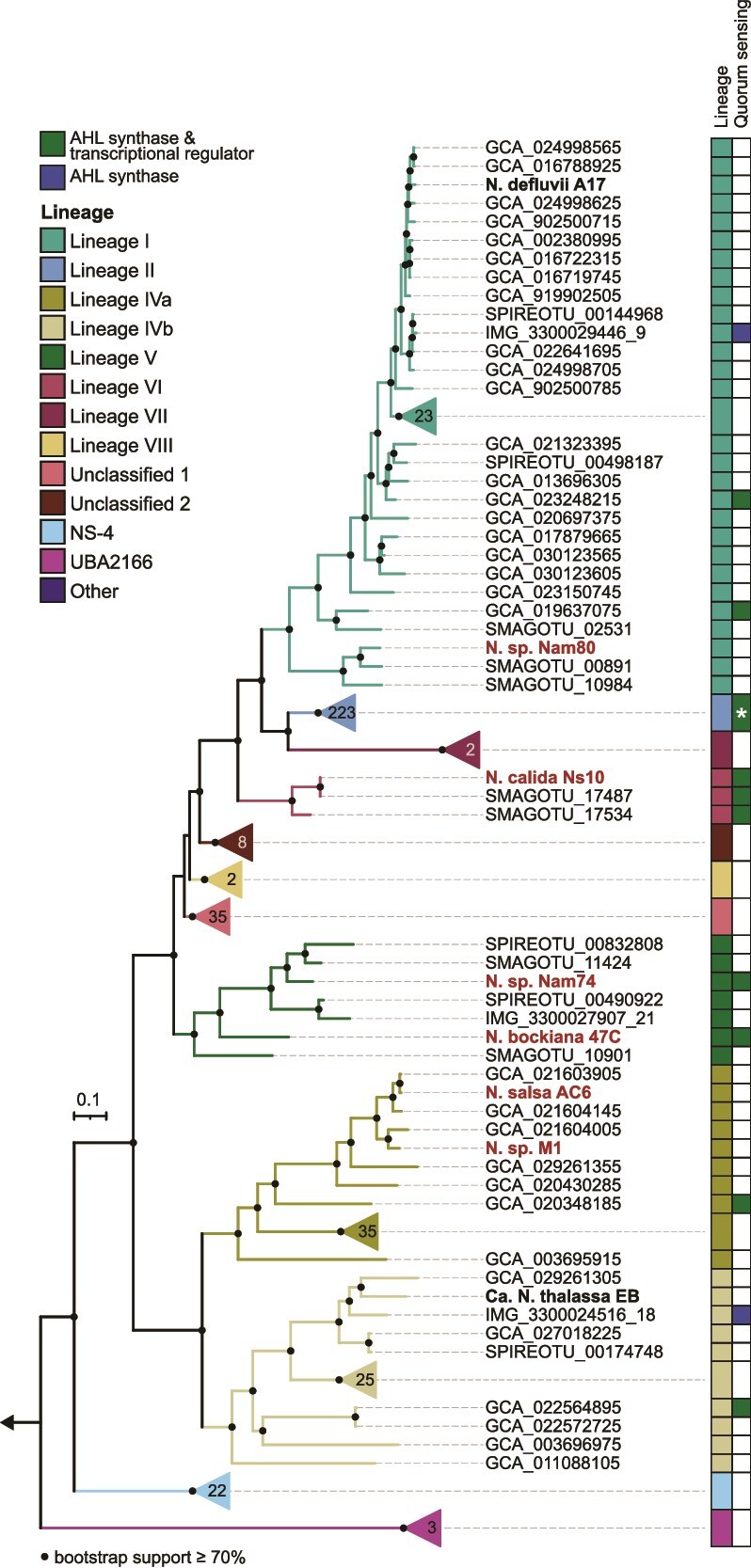
**Distribution of AHL synthase and quorum sensing transcriptional regulator genes in the *Nitrospirales* genomes.** The number on the collapsed clades indicates the number of genomes in this group. Quorum sensing genes were found in 67.3% of lineage II genomes, as indicated by the ^*^, but are not shown here to focus on the other lineages where quorum sensing has not yet been described. Groups with no quorum sensing genes detected were also collapsed. The phylogenomic tree is the same as in Fig. 1, with black circles indicating bootstrap support ≥70% of 1000 ultrafast bootstrap replicates. The scale bar represents 10% sequence divergence. Tree clades are colored according to the lineage classification. Newly sequenced genomes are labeled in bold red, genomes of cultivated *Nitrospirales* in bold black font. GTDB representatives belonging to orders other than the *Nitrospirales* within the class *Nitrospiria* were used as the outgroup, as indicated by the arrow.

We found LuxI homologs in more than a third of the genomes analysed (36.04%), of which most also possess LuxR. Most of these occur in lineage II genomes, predominantly in comammox organisms. Although LuxR homologs were also identified in many lineage I genomes lacking LuxI, it is unclear whether these regulators can respond to AHL produced by other organisms. The two closed lineage V genomes (*Nitrospira* sp. Nam74 and *Ca*. N. bockiana) and all three available lineage VI genomes (including *Ca*. N. calida) possess both AHL synthase and regulator genes, thereby expanding putative quorum sensing beyond lineage II (Fig. 5). In addition, two lineage IV genomes possess AHL synthase genes located on short contigs. These genes clustered with *Alphaproteobacteria* in phylogenetic analyses*,* whereas surrounding genes are homologous to *Nitrospirales* sequences (not shown). Thus, although the possibility of assembly and binning errors in these MAGs cannot be excluded, horizontal gene transfer might be the origin of these genes in the lineage IV genomes.

### Comparison of the NXR-containing *Nitrospirales* NS-4 to non-nitrite-oxidizing *Nitrospiria* clades

To assess the potential metabolic changes associated with the acquisition of nitrite oxidoreductase and the putative switch to a nitrite-oxidizing lifestyle, we compared the available genomes of the most deep-branching clade encoding nitrite oxidoreductase (NS-4) with their closest relatives (UBA2166) lacking these key functional genes. To maximize the information associated with the transition to the nitrite oxidizer physiology, we retrieved the genomes from the uncharacterized NXR-negative JACQCE01 and JACQBZ01 orders, as well as the uncharacterized UBA2166 and NS-4 families within the *Nitrospirales* order from GlobDB (see methods; Fig. S16).

The UBA2166 family is the only clade in the *Nitrospirales* order lacking NXR, but in phylogenomic analysis, the available genomes form a long branch within the *Nitrospirota* (Fig. S16). A comparative analysis with JACQCE01, JACQBZ01, and NS-4 genomes indicates that the UBA2166 species possess 544 family-specific genes (Figs. S16 and S17; Table S9), indicating that this family is not the ideal outgroup to investigate the transition to the nitrite-oxidizing phenotype. The genes acquired by the UBA2166 organisms include the sulfite transporter TauE, two molybdoenzymes of the sulfite oxidase superfamily, and, as previously observed, genes homologous to *soxYZ* [131]. However, the remaining *sox* genes required for thiosulfate oxidation were not found in these genomes. Like the rest of the *Nitrospirales*, the UBA2166 genomes encode the capability for CO_2_ fixation using the rTCA cycle. Using marker genes, genomes from the UBA2166 family were primarily detected in metagenomes from marine systems, frequently in marine sediments or at hydrothermal vents (Table S10). Although a full analysis of the metabolic capabilities of the UBA2166 family is beyond the scope of this study, the observations listed above indicate a possible role in sulfur cycling.

In our comparative analysis, we identified 343 genes specific to NS-4 relative to UBA2166, JACQCE01, and JACQBZ01 (Table S11). We then assessed the presence of those genes across the NXR-encoding *Nitrospirales* genomes presented here. We found that 140 genes are conserved in the majority (>69%) of these genomes (Table S12) and propose that these are diagnostic for the nitrite-oxidizing metabolism in the order *Nitrospirales*. Among these genes are the NXR subunits, as well as the TorD-like maturase that could be specific for the molybdenum-containing NxrA subunit. Several of these genes are part of a conserved cluster (NITMOv2_3614 - NITMOv2_3631 in *N. moscoviensis*) encoding, besides the NxrC, TorD, and several proteins of unknown function, also two putative pentaheme cyt. *c* proteins and a regulatory two-component system. These proteins were found to be expressed in *N. moscoviensis* under nitrite-oxidizing growth conditions [73], indicating their importance for the nitrite-oxidizing phenotype. Furthermore, the gene set contained several additional cyt. *c* proteins that could play a role in the electron transport chain, as well as the NO-forming nitrite reductase (NirK). We also identified the chlorite dismutase, which has been noted to be a feature of diverse nitrite-oxidizing bacteria [132] and was previously characterized in *Nitrospira defluvii* [133, 134]. Many of the identified proteins have no known function, suggesting that this gene list provides useful targets for future analysis and characterization to better understand the genesis of the nitrite-oxidizing lifestyle that is characteristic of present-day *Nitrospirales*. Although this comparative approach provided first insights into the transition of *Nitrospirales* to a nitrite-oxidizing lifestyle, future studies are needed to elucidate the evolutionary history and the underlying mechanisms.

## Conclusions

Our study has significantly advanced our understanding of the genomic and metabolic landscape of *Nitrospirales* by revealing new insights into their phylogenetic and functional diversity. The inclusion of genomes of lineages without previously genome-sequenced representatives and the newly proposed lineage VIII underscores the expanding complexity within this fascinating nitrifier group. Moreover, the discrepancies between historically established lineage classifications and the GTDB taxonomy highlight the importance of revising current taxonomic frameworks. The identified differences between 16S rRNA gene-based and phylogenomic trees illustrate the drawbacks and limitations of single-gene markers for taxonomic classification. The lack of genomes and cultures from the 16S rRNA gene-based lineage III also demonstrates the still underexplored diversity within the *Nitrospirales* to be addressed in future diversity studies.

In our analyses, we focused on the modularity and variability of the respiratory chain and the assimilatory nitrogen metabolism, which reflect the complex evolutionary history and ecological flexibility of *Nitrospirales*, enabling adaptation to varying and fluctuating environmental conditions. The finding of quorum sensing mechanisms across multiple lineages suggests that intercellular communication may be more widespread and integral to the ecological success of the *Nitrospirales* than previously recognized. Lastly, we identified a set of conserved genes in the extant *Nitrospirales* that are likely associated with the transition to their nitrite-oxidizing physiology.

## Supplementary Material

04_Kop_Koch_2025_ISMEJ_SupplementaryMaterials_Rev1_wraf151

Kop_Koch_2025_Nitrospirales_SupplTabl-01_wraf151

Kop_Koch_2025_Nitrospirales_SupplTabl-02_wraf151

Kop_Koch_2025_Nitrospirales_SupplTabl-03_wraf151

Kop_Koch_2025_Nitrospirales_SupplTabl-04_wraf151

Kop_Koch_2025_Nitrospirales_SupplTabl-05_wraf151

Kop_Koch_2025_Nitrospirales_SupplTabl-06_wraf151

Kop_Koch_2025_Nitrospirales_SupplTabl-07_wraf151

Kop_Koch_2025_Nitrospirales_SupplTabl-08_wraf151

Kop_Koch_2025_Nitrospirales_SupplTabl-09_wraf151

Kop_Koch_2025_Nitrospirales_SupplTabl-10_wraf151

Kop_Koch_2025_Nitrospirales_SupplTabl-11_wraf151

Kop_Koch_2025_Nitrospirales_SupplTabl-12_wraf151

## Data Availability

All genomic sequencing data generated and analysed during the current study are available at the European Nucleotide Archive, https://www.ebi.ac.uk/ena/browser/view/PRJEB85292.
