# Supplementary material for: Comparative genome analysis reveals broad phylogenetic and functional diversity within the order Nitrospirales
Source: ISME J. 2025 Jul 22;19(1):wraf151. doi: 10.1093/ismejo/wraf151 (PMC12342949; doi:10.1093/ismejo/wraf151)
Supplement: 04_Kop_Koch_2025_ISMEJ_SupplementaryMaterials_Rev1_wraf151 [file 04_kop_koch_2025_ismej_supplementarymaterials_rev1_wraf151.pdf]

Comparative genome analysis reveals broad phylogenetic and functional diversity within the order *Nitrospirales*

Linnea F.M. Kop[1,2,3,#], Hanna Koch[1,4,#], Daan Speth[2], Claudia Lüke[1], Eva Spieck[5], Mike S.M. Jetten[1], Holger Daims[2,6], Sebastian Lücker[1,*]

[1] Department of Microbiology, Radboud Institute for Biological and Environmental Sciences, Radboud University, Heyendaalseweg 135, 6525 AJ, Nijmegen, the Netherlands

[2] Division of Microbial Ecology, Centre for Microbiology and Environmental Systems Science, University of Vienna, Djerassiplatz 1, 1030 Vienna, Austria

[3] Doctoral School in Microbiology and Environmental Science, University of Vienna, Djerassiplatz 1, 1030 Vienna, Austria

[4] Bioresources Unit, Center for Health & Bioresources, AIT Austrian Institute of Technology GmbH, Konrad-Lorenz-Straße 24, 3430 Tulln an der Donau, Austria

[5] Department of Microbiology and Biotechnology, University of Hamburg, Ohnhorststr. 18 22609 Hamburg, Germany

[6] The Comammox Research Platform, University of Vienna, Djerassiplatz 1, 1030 Vienna, Austria

[#] These authors contributed equally.

* Corresponding author: Sebastian Lücker, s.luecker@science.ru.nl

Supplementary Materials

This document contains the Supplementary text and Supplementary Figures S1-S17, as well as the descriptions for Supplementary Tables S1-S12, which are provided as separate files.

All Supplementary Figures are available as high-quality PDF files at:
https://doi.org/10.6084/m9.figshare.c.7705802.v2

Supplementary Materials and Methods

DNA isolation

For DNA isolation of *Ca.* N. calida and *Ca.* N. bockiana, 100 ml of each culture were harvested by filtering onto 1 µm polycarbonate Whatman Cyclopore PC 47 mm filters (Cytiva, USA). Similarly, 35-80 ml biomass of the *Nitrospira* spp. Nam74, Nam80, Kam-Ns4a, and LUA16 enrichments were filtered onto 0.2 µm polycarbonate Whatman Cyclopore PC 47mm filters. All filters were stored at -20 °C. DNA isolation from the filtered biomass was performed using the DNeasy Blood and Tissue kit (Qiagen, United Kingdom) according to Renshaw and coworkers [1] and the manufacturer's instructions, but with a slight modification: before lysozyme treatment, the filters were submerged in 540 µl ATL buffer and subsequently incubated at -80 °C for ≥30 min.

For *Ca.* N. salsa and *Nitrospira* sp. Ecomares 2.1, 25-100 ml of biomass was harvested via centrifugation. The resulting cell pellets were resuspended in 1x PBS (pH 7.4), and a proteinase K treatment at 56 °C for 10 min (12 mAU) was performed. All subsequent steps of DNA isolation were done using the DNeasy Blood and Tissue (Qiagen, USA) kit according to the manufacturer's instructions. The DNA of *Nitrospira* sp. M1 was isolated using the PowerSoil DNA isolation kit (MO BIO Laboratories, USA) according to the manufacturer's instructions with slight modifications according to Spieck and coworkers [2].

The DNA concentrations for all samples were measured by Qubit v2.0 using the Qubit HS dsDNA kit (Thermo Fisher Scientific, USA). All DNA samples were sequenced using short and long-read sequencing technologies as described below.

Short-read Illumina sequencing

For Illumina sequencing, libraries were prepared using ≥1 ng DNA per sample and the Nextera XT kit (Illumina, USA) according to the manufacturer's protocol. All steps of library preparation were performed as described by Keuter and coworkers [3]. The pooled libraries were sequenced paired-end (2 x 300 bp) on the MiSeq system using MiSeq reagent kit v3 (Illumina). Prior to assembly and binning, the raw reads were trimmed using bbduk within bbtools v.37.76 (https://jgi.doe.gov/data-and-tools/bbtools) with the following settings: k=23, mink=11, hdist=1, ktrim=r, tbo, qtrim=rl, maq=20, maxns=0, minlen=150, tossjunk=t and trimq = 17.

Long-read Nanopore sequencing

For long-read MinION (Oxford Nanopore Technologies, UK) sequencing, libraries were constructed using different amounts of input DNA. The used DNA quantities, Ligation Sequencing kits 1D, and Native Barcoding Expansion kits are summarized in Table S2. The kits were used according to the manufacturer's protocol (Oxford Nanopore Technologies). After end repair and dA-tailing, purification, quantification, and adapter ligation as described in previous studies [3, 4], the libraries were purified using AMPure XP beads (Beckman Coulter Life Sciences, USA) and loaded on a Flow cell (FLO-MIN106, Oxford Nanopore Technologies). The base calling softwares and models used are also summarized in Table S2.

Hybrid assemblies using short- and long-read sequencing data

Different hybrid assembly strategies were chosen for obtaining *Nitrospira* MAGs.
The sequencing adapters were removed from the NanoPore reads using porechop (v.0.2.3_seqan2.1.1; https://github.com/rrwick/Porechop) with default settings. For *Ca.* N. calida, *Ca.* N. bockiana, and *Ca.* N. salsa, the trimmed Illumina and NanoPore reads were assembled using the hybrid assembler unicycler (v.0.4.4) [5] with default settings and --start_gene_id 60 --start_gene_cov 80, providing the chromosomal replication initiator protein DnaA of *N. moscoviensis* (UniProt: A0A0K2G696) as reference. This approach resulted in circular genomes of *Ca.* N. calida and *Ca.* N. bockiana. As this approach did not yield a complete genome for *Ca.* N. salsa, the hybrid assembly was manually binned using anvi'o (v.6) [6] using the anvi'o interactive interface and the bin refinement tool 'anvi-refine' to further improve the quality of the *Ca.* N. salsa MAG. The genome of *Nitrospira* sp. Ecomares 2.1 was also assembled using unicycler (v.0.4.7) [5] with default settings, but the circular genome was manually centered at the DnaA sequence based on a prokka (v.1.12-beta) annotation [7].
For the enrichment cultures *Nitrospira* sp. Kam-Ns4a, *Nitrospira* sp. Nam80, *Nitrospira* sp. M1, and *Nitrospira* sp. LUA16, the trimmed NanoPore reads were assembled using flye (v.2.9-b1768) with following settings: -g 5m, --meta. The trimmed NanoPore reads for *Nitrospira* sp. Nam74 were assembled using Canu (v.1.8) [8] with the following parameters: genomeSize=6m. The obtained assemblies were polished using minimap2 (v.2.16-r922) [9] to map long reads onto the assembly and Racon v1.3.1 [10] for polishing with default settings. Subsequently, the *Nitrospira* MAGs were identified in the metagenomic assemblies using GDTB-Tk, and the trimmed Illumina reads from the respective sample were mapped onto these using bbmap.sh v.37.76 (sourceforge.net/projects/bbmap/) with a minimum

identity of 80%. The Illumina reads that mapped onto the *Nitrospira* MAGs were used for the hybrid assembly using unicycler (v.0.4.4) [5] with the closed *Nitrospira* MAG as existing long read assembly and the settings described above.

Urease phylogeny

The amino acid sequences of the urease alpha subunit (UreC) were extracted from the manually curated DRAM annotations of the *Nitrospirales* genomes. A reference dataset of UreC sequences was assembled by downloading UreC sequences from NCBI with a length of 500-650 amino acids, excluding eukaryotic or partial sequences and sequences for the homologous amidohydrolases (YtcJ). The downloaded sequences were clustered at 90% identity using usearch (v11.0.667_i86linux32) [11]. The reference dataset was manually curated to remove misannotated and duplicated sequences, as well as sequences with frameshifts, gaps, or insertions by inspecting the muscle (v3.8.31) [12] alignments and calculating neighbor-joining trees in ARB (arb-7.0) [13], with additional information from InterProScan [14]. The *Nitrospirales* UreC sequences were then aligned to the reference dataset using muscle (v3.8.31) [12]. Alignment positions with gaps in more than 5% of the sequences were removed using trimAl (v1.4.rev22, -gt 0.95) [15]. IQ-Tree (v1.6.12) with 1000 ultra-fast bootstrap replicates was used to construct the phylogenetic tree, with the Q.pfam+I+G4 model identified by ModelFinder [16, 17].

Cyanase phylogeny

Cyanase (CynS) sequences were retrieved from genomes included in the GlobDB (https://globdb.org/). A previously published CynS amino acid sequence dataset [18] was used as seed to retrieve a comprehensive CynS dataset (10,760 sequences) from bacteria and archaea, using an alignment score ratio approach as described previously [19, 20]. CynS sequences originating from *Nitrospira* GlobDB genomes were removed from the dataset, and the CynS sequences encoded in the genomes analyzed in this study (which have a more stringent quality cutoff) were added.

To assess the position of the *Nitrospirales* CynS sequences in this global dataset, we aligned the sequences using muscle (v5.1) and calculated a phylogeny using FastTree (version 2.1.11) [21]. Based on this phylogeny, a subset of sequences representing the phylogenetic neighborhood of the two clades containing *Nitrospirales* sequences was selected. This subset of sequences was aligned with muscle (v5.1), and this alignment was used to calculate a phylogenetic tree using IQ-Tree (v2.3.5) [22], with LG+F+I+R7 chosen as the best model by

ModelFinder [16], constrained to using the models LG, JTT, WAG, LG4M, and LG4X. 1000 ultrafast bootstrap replicates were generated using UFBoot2 [23].

Nitrite oxidoreductase phylogeny

Sequences of the catalytic subunit of nitrite oxidoreductase (*nxrA*) were retrieved from the *Nitrospirales* genomes using a protein alignment score ratio (PASR) approach with confirmed NxrA sequences as seed. The PASR approach is based on Rasko and coworkers, and the scripts used for calculation are available under https://github.com/dspeth/PASR [19, 20]. The retrieved 399 sequences were aligned using muscle (v5.1) [24] and a phylogenetic tree was calculated using IQ-Tree (v2.3.5) [22], with LG+R6 chosen as the best model by ModelFinder [16], constrained to using the models LG, JTT, WAG, LG4M, and LG4X. 1000 ultrafast bootstrap replicates were generated using UFBoot2. As the *Nitrospirales* NxrA are known to form a distinct clade separate from other related sequences (e.g., *Nitrospina* or *Brocadiaceae* NxrA), and the sequences in the NS-4 group were found to be basal to the *Nitrospirales* clade, we rooted the NxrA tree using the NS-4 sequences.

Quinol-oxidizing *bd*-type O_2 reductase (CydA) phylogeny

CydA sequences encoding quinol-oxidizing *bd*-type O_2 reductases from the *Nitrospirales* genomes were aligned to the sequences from the multiple sequence alignment (MSA2) by Murali et al. [25]. Alignment trimming and tree calculation were performed as described above for the UreC tree with trimAl and IQ-Tree using the VT+F+G4 model identified by ModelFinder.

Supplementary Results and Discussion

Completeness and redundancy estimates

In our analysis, we found a profound discrepancy between the expected quality of circular/closed genomes and the estimates provided by CheckM [26]. Of 18 closed *Nitrospira* genomes analyzed - including both newly sequenced and previously published genomes - none were rated as complete by CheckM, and all were reported as contaminated (Figure S5, Table S1). The average completeness and contamination of these genomes were 96.42% and 3.42%, respectively. Three genomes had contamination levels above 5%, a threshold commonly used to define high quality genomes. These results suggest that reliance on single-copy marker genes may not always accurately reflect the quality of *Nitrospirales* genomes due to the absence of one or more of the core single-copy genes defined in CheckM, and the

presence of paralogs. Therefore, we also determined the estimated completeness and contamination of the genomes using CheckM2 (Figure S5), which improved the overall estimates. However, several genomes were still estimated to be contaminated, e.g., *Nitrospira* sp. LUA16 was estimated to have 6.56% contamination. In conclusion, although overall quality estimates have improved, these still do not always accurately reflect the quality of genomes.

Urease accessory proteins

The accessory proteins encoded by the genes *ureE*, *ureF*, *ureG*, and *ureD* have been shown to be important for the incorporation of nickel (Ni^{2+}) into the urease [27, 28] and experimental evidence suggests that *ureF*, *ureG*, and *ureD* are essential for urease activity in *Klebsiella aerogenes* and *Helicobacter pylori* [27–29]. In addition, *ureE* knockout mutants lose 99-100% of their urease activity, indicating the gene's necessity for urease maturation [28, 30]. Whereas almost all genomes with a *ureC* also contain *ureFGD*, *ureE* is absent from most genomes except those encoding a group 3 UreC and one genome with a group 1b UreC (GCA_015904025.1; Figure 3, Figure S13). These genomes likely acquired the urea degradation gene cluster from a different source than the majority of *Nitrospirales*.
Despite the absence of *ureE* in *N. moscoviensis*, its ureolytic activity was experimentally demonstrated [31]. Here, it was speculated that the hydrogenase accessory proteins (HypAB) might substitute UreE as the nickel-binding metallochaperone, but a distribution analysis of the *hypAB* genes in *Nitrospirales* showed no correlation between their presence and the absence of UreE in urease-encoding genomes (Figure S13).
Zambelli et al. [32] found that among 284 closed genomes from diverse phylogenetic groups, 13% lacked UreE, suggesting the existence of an alternative mechanism for nickel loading of the urease in these organisms, which is likely also the case in *Nitrospirales* lacking UreE. Similarly, no UreE homolog has been identified in plants. Plant UreG contains a histidine (His)-rich N-terminus, which may fulfill the nickel-binding role observed in the His-rich N-terminus of bacterial UreE [33]. However, UreE mutants lacking this N-terminus still exhibited high urease activity, suggesting a role in nickel storage rather than metallocenter assembly [34]. Nevertheless, we found that the majority of *Nitrospirales* UreG sequences have a His-rich N-terminus, which is lacking in those *Nitrospirales* genomes that encode a group 3 UreC and UreE. Therefore, it is tempting to speculate that the UreG proteins with the His-rich N-terminus play a role in nickel binding in UreE-deficient *Nitrospirales*.

Supplementary Figures

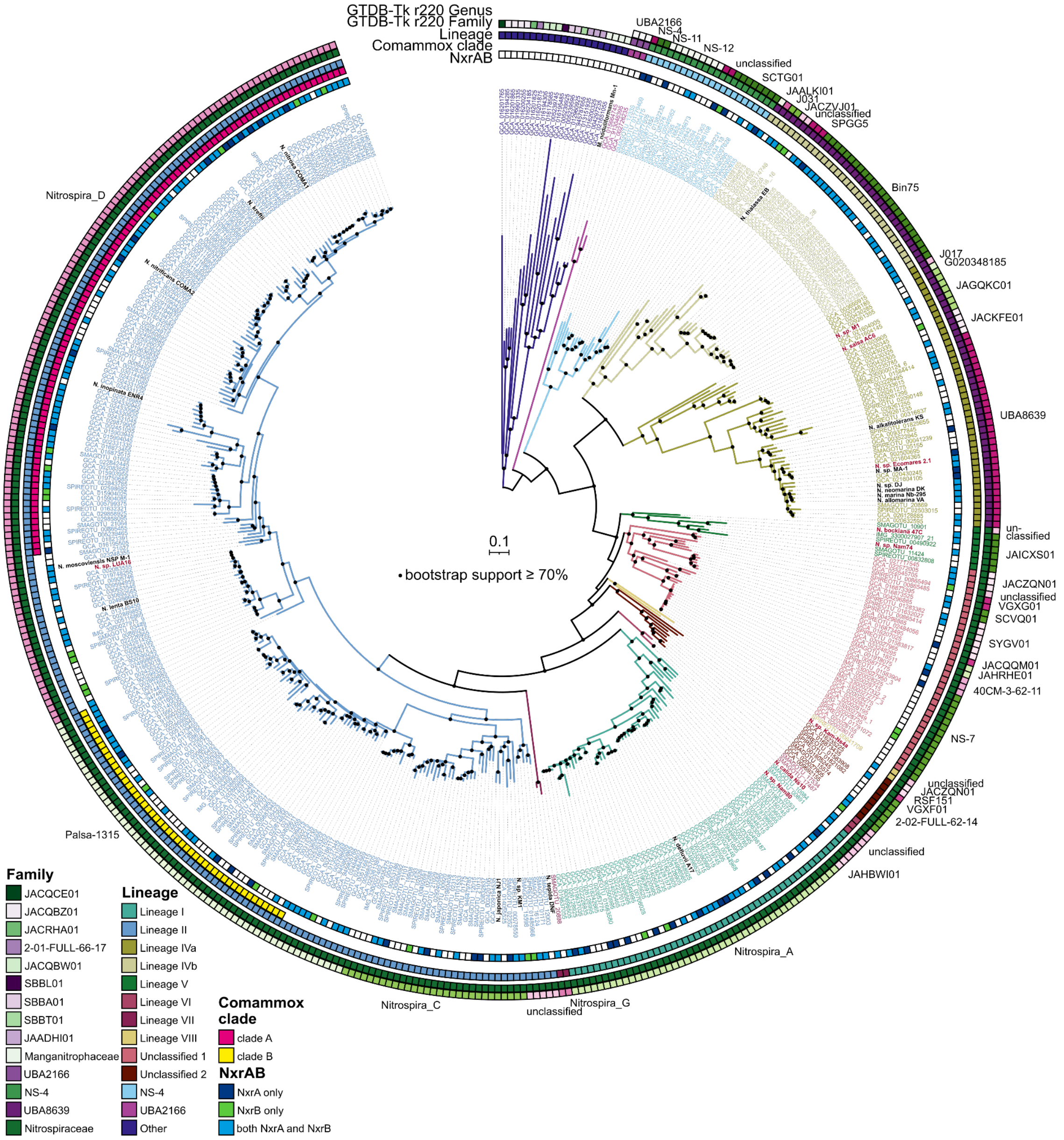

Figure S1. Phylogenomic tree of non-redundant *Nitrospirales* genomes (≥75% estimated completeness, ≤10% estimated redundancy) based on the concatenated alignment of 71 core proteins. The phylogenomic tree is the same as in main text Figure 1, but includes all names. Black circles indicate bootstrap support ≥70% of 1000 ultrafast bootstrap replicates. The scale bar represents 10% sequence divergence. Tree branches are colored according to the lineage classifications. Newly sequenced genomes are labeled in bold red, genomes of cultivated *Nitrospirales* in bold black font. The prefix '*Candidatus*' was omitted for brevity; for details, see Table S1. Additional information on the presence of NxrAB subunits, and comammox clade, lineage, and GTDB-Tk classifications (r220) are shown in the rings surrounding the tree. GTDB representatives belonging to orders other than the *Nitrospirales*

within the class *Nitrospiria* are labeled as ‘Other’, and their GTDB-Tk genus classifications were omitted for clarity.

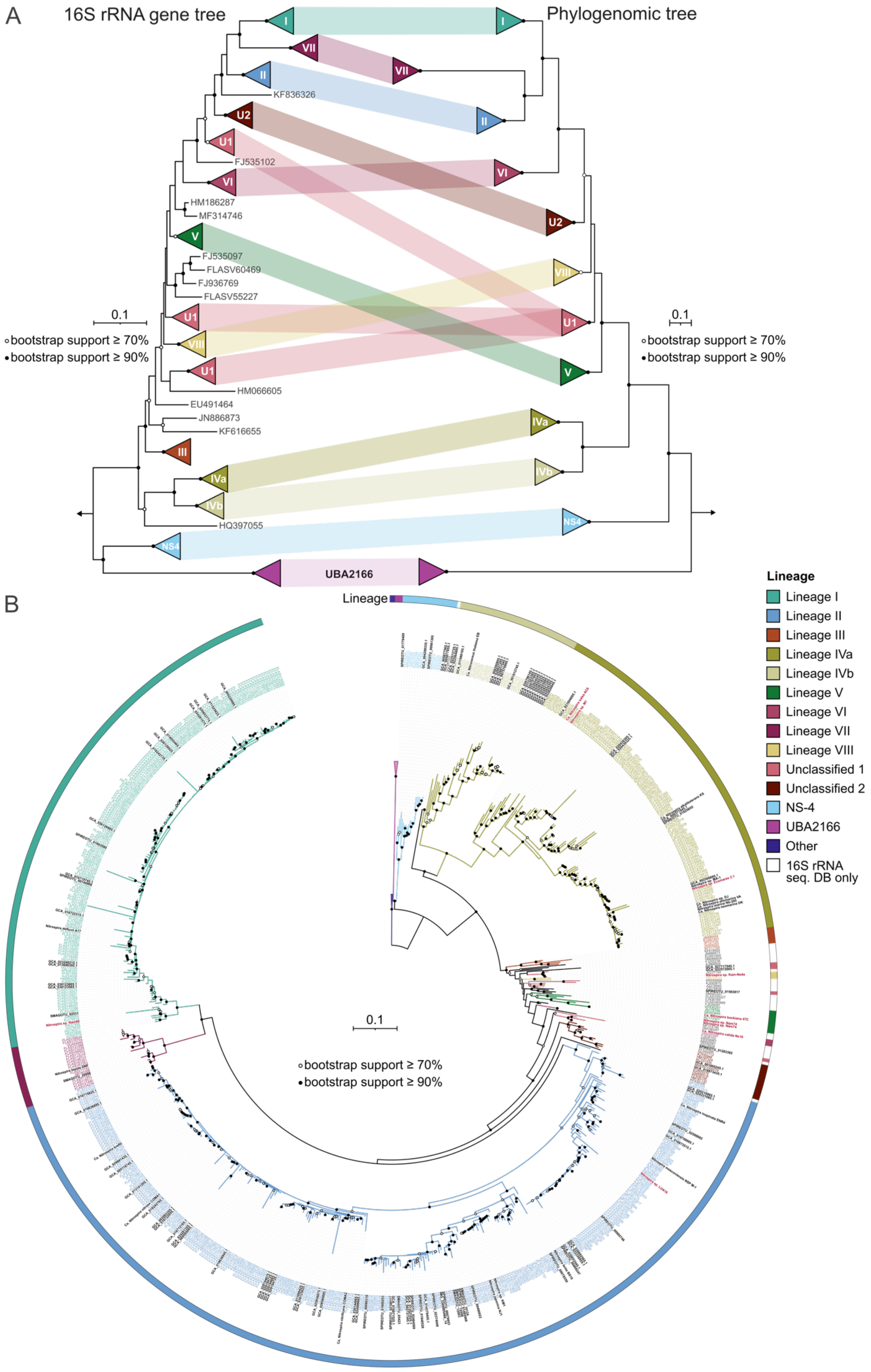

A
16S rRNA gene tree
Phylogenomic tree
I
VII
II
KF836326
U2
U1
FJ535102
VI
HM186287
MF314746
V
FJ535097
FLASV60469
FJ936769
FLASV55227
VIII
HM066605
EU491464
JN886873
KF616655
III
IVa
IVb
HQ397055
NS4
UBA2166
0.1
∘bootstrap support ≥ 70%
•bootstrap support ≥ 90%
B
Lineage
Lineage I
Lineage II
Lineage III
Lineage IVa
Lineage IVb
Lineage V
Lineage VI
Lineage VII
Lineage VIII
Unclassified 1
Unclassified 2
NS-4
UBA2166
Other
16S rRNA seq. DB only

Figure S2. 16S rRNA gene-based phylogeny. **A)** Comparison of 16S rRNA gene-based and whole-genome phylogenies of the non-redundant *Nitrospirales* genomes. The phylogenomic tree is the same as in main text Figure 1. Tree branches and labels are colored according to the lineage classifications. GTDB representatives belonging to orders other than the *Nitrospirales* within the class *Nitrospiria* were used as the outgroups for each tree, as indicated by the arrows. **B)** A detailed view of the 16S rRNA gene-based phylogeny, with tree branches and labels colored according to lineage classifications. White and filled circles indicate, respectively, bootstrap support ≥ 70% and ≥ 90% of 1000 ultrafast bootstrap replicates. Scale bars represent 10% sequence divergence. Tree branches and labels are colored according to the lineage classifications. Lineage classifications are shown in the ring surrounding the tree, with unclassified reference sequences marked in white. The 16S rRNA gene sequences extracted from the analyzed *Nitrospirales* genomes are shown in bold black font, with the exception of the newly sequenced genomes, which are shown in bold red.

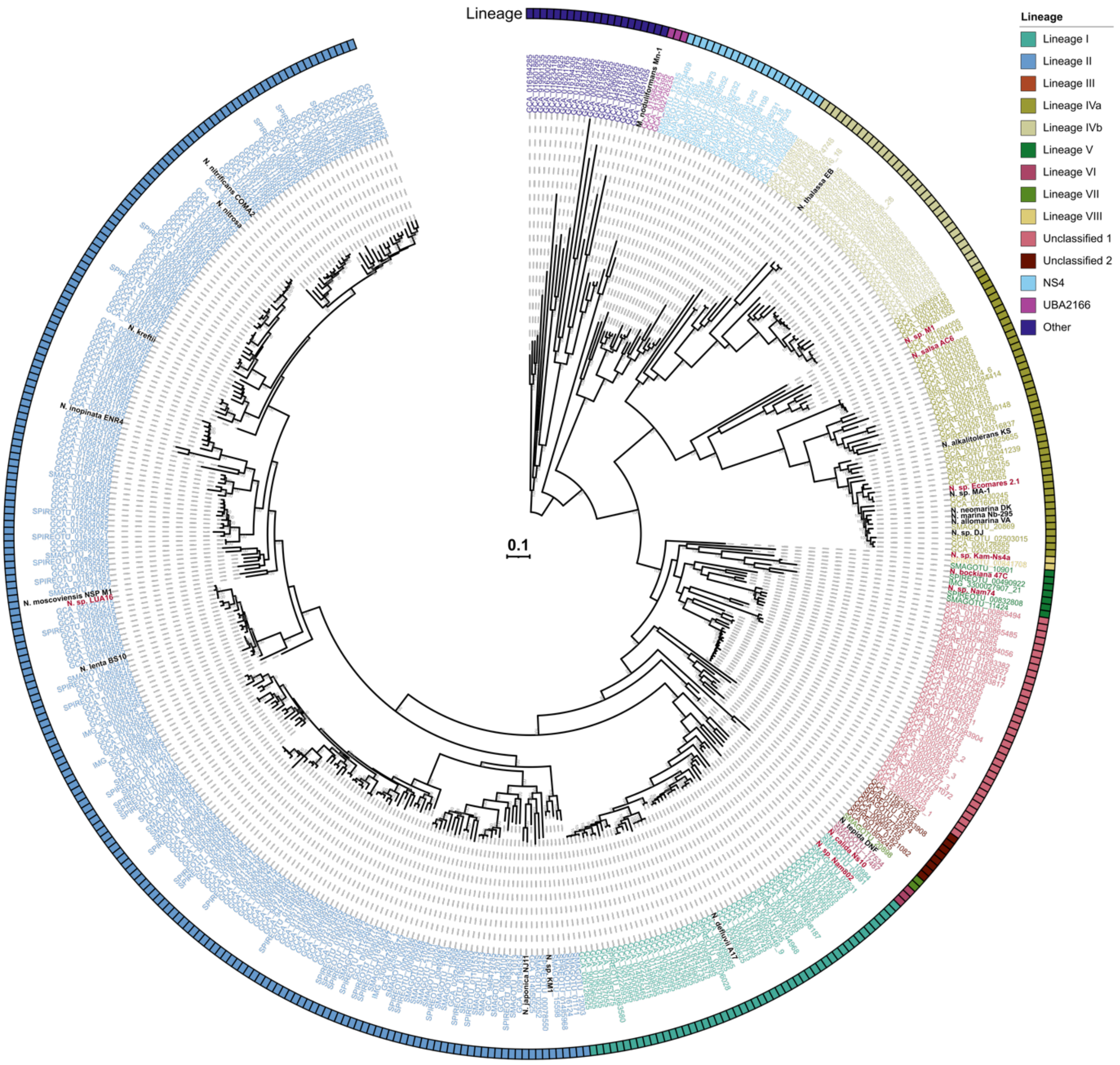

Figure S3. Phylogenomic tree of non-redundant *Nitrospirales* genomes (≥75% estimated completeness, ≤10% estimated redundancy) based on the concatenated alignment of 120 core proteins identified by GTDB-Tk. Bootstrap support ≥90% of 1000 ultrafast bootstrap replicates is shown in grey font. The scale bar represents 10% sequence divergence. Tree branches are colored according to the lineage classifications. Newly sequenced genomes are labeled in bold red, genomes of cultivated *Nitrospirales* in bold black font. The prefix '*Candidatus*' was omitted for brevity; for details, see Table S1. GTDB representatives belonging to orders other than the *Nitrospirales* within the class *Nitrospiria* are labeled as 'Other'.

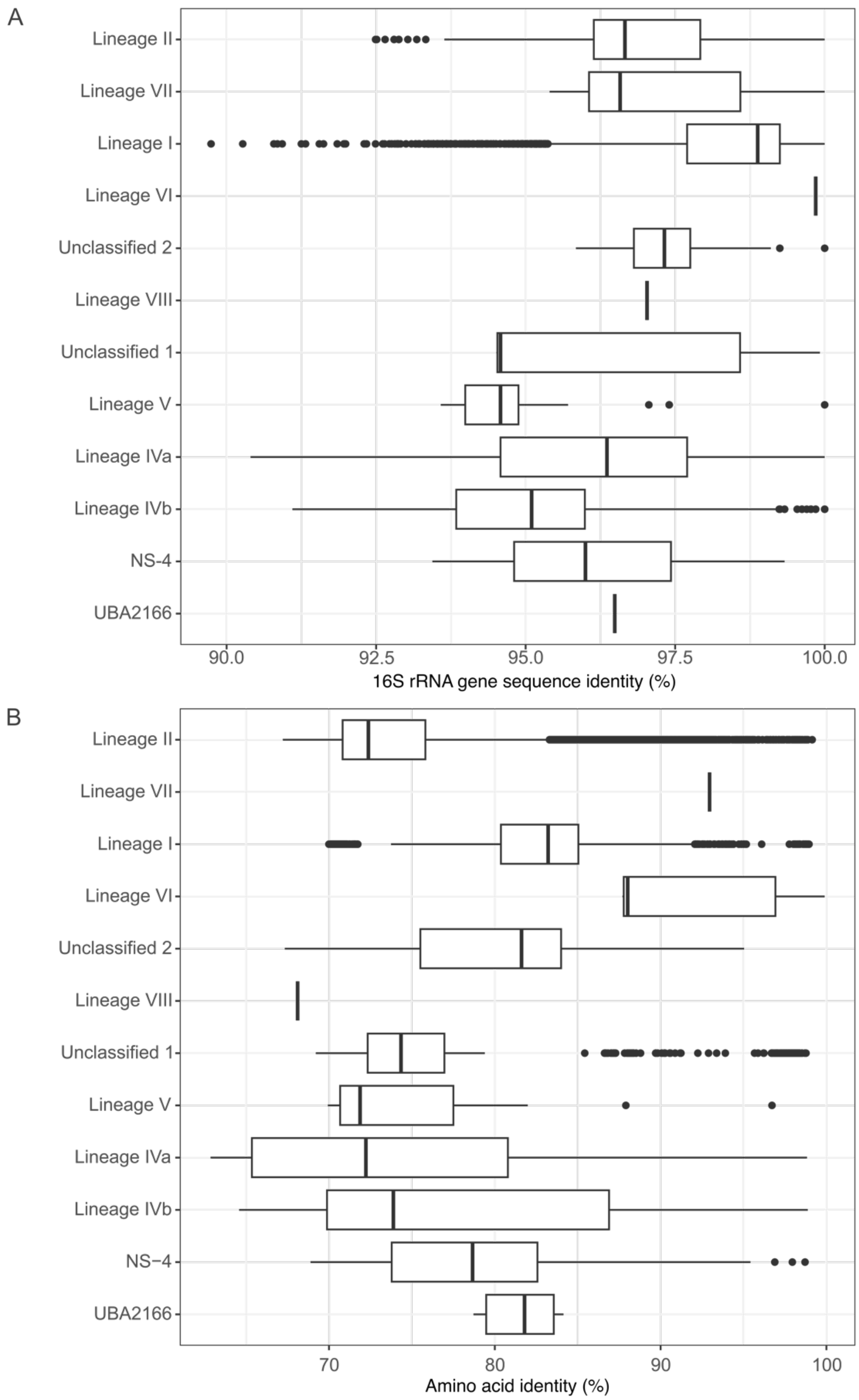

Figure S4. Sequence identities within lineages. **A)** 16S rRNA gene sequence identities and **B)** average amino acid identities (%) within *Nitrospirales* lineages. The side borders of the boxes represent the 25th and 75th percentiles, the thick lines represent the medians. The whiskers extend to the largest and smallest value no further than 1.5 times the interquartile range from the quartile border. Outliers are shown as dots.

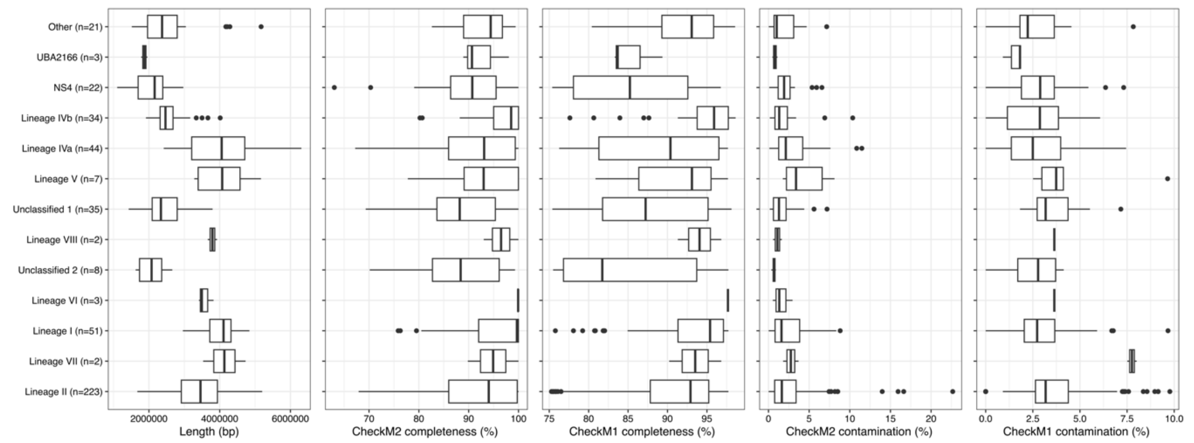

Figure S5. Genome length (bp) and estimated completeness and contamination (%) of the non-redundant *Nitrospirales* genomes. GTDB representatives belonging to orders other than the *Nitrospirales* within the class *Nitrospiria* are labeled as 'Other'. The side borders of the boxes represent the 25th and 75th percentiles, the thick lines represent the medians. The whiskers extend to the largest and smallest value no further than 1.5 times the interquartile range from the quartile border. Outliers are shown as dots.

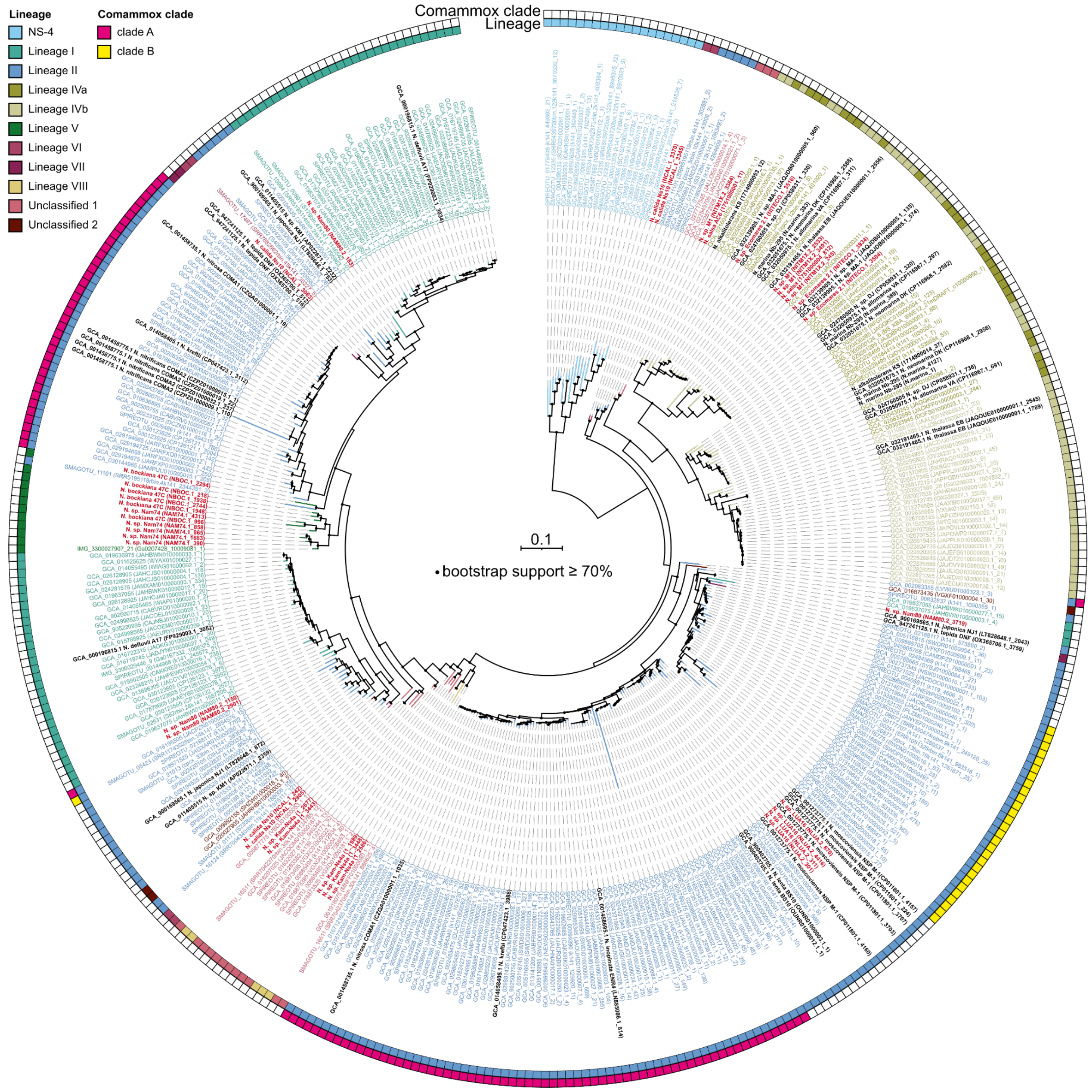

Figure S6. NxrA-based phylogeny of the non-redundant *Nitrospirales* NxrA sequences. Tree branches and labels are colored according to the lineage classifications. Newly sequenced genomes are labeled in bold red, genomes of cultivated *Nitrospirales* in bold black font. In addition, information on lineage and comammox clade classifications is shown in the rings surrounding the tree. Black circles indicate bootstrap support ≥70% of 1000 ultrafast bootstrap replicates. Scale bars represent 10% sequence divergence. The tree was rooted using the NS-4 NxrA sequences. The prefix '*Candidatus*' was omitted from the species names for brevity; for details, see Table S1.

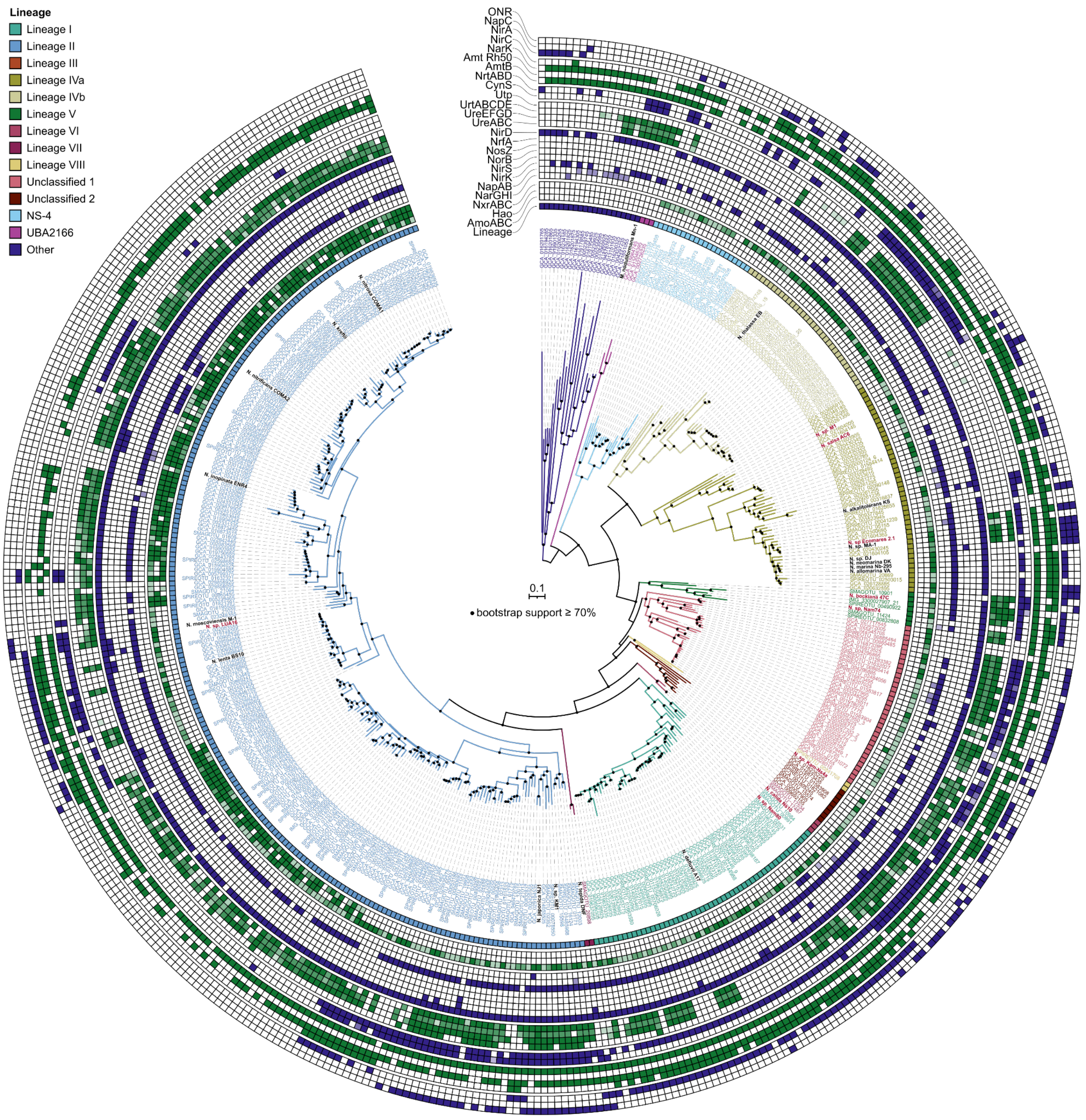

Figure S7. Heatmap showing the distribution and completeness of genes involved in nitrogen metabolism in non-redundant *Nitrospirales* genomes, shown in circles around the same phylogenetic tree as in Figure 1. Completeness of complexes is indicated by the color shade. Black circles indicate bootstrap support ≥70% of 1000 ultrafast bootstrap replicates. The scale bar represents 10% sequence divergence. Tree branches and labels are colored according to the lineage classifications. Newly sequenced genomes are labeled in bold red, genomes of cultivated *Nitrospirales* in bold black font. The prefix '*Candidatus*' was omitted from the species names for brevity; for details, see Table S1. Abbreviations: AmoABC = ammonia monooxygenase; Hao = hydroxylamine dehydrogenase; NxrABC = nitrite oxidoreductase; NarGHI = nitrate reductase; NapAB = periplasmic nitrate reductase; NirK =

NO-forming copper-containing nitrite reductase; NirS = NO-forming cytochrome cd_1-containing nitrite reductase; NorB = nitric oxide reductase subunit B; NorC = nitric oxide reductase subunit C; NosZ = nitrous oxide reductase; NrfA = respiratory nitrite reductase (cytochrome *c*-552); NrfH = respiratory nitrite reductase (cytochrome *c*) small subunit; NirB = nitrite reductase (NADH) large subunit; NirD = nitrite reductase (NADH) small subunit; NirA = assimilatory nitrite reductase; NapC = cytochrome *c*-type protein; ONR = putative octaheme cytochrome *c* nitrite reductase; NarK = nitrate transporter; NirC = nitrite transporter; UreABC = urease; UreEFGD = urease accessory proteins; UrtABCDE = urea ABC transporter; Utp = urea transporter (UT) family protein; CynS = cyanase; NrtABD = nitrate/nitrite/cyanate transporter; AmtB = ammonia transporter; Amt Rh50 = Rh50 ammonia transporter.

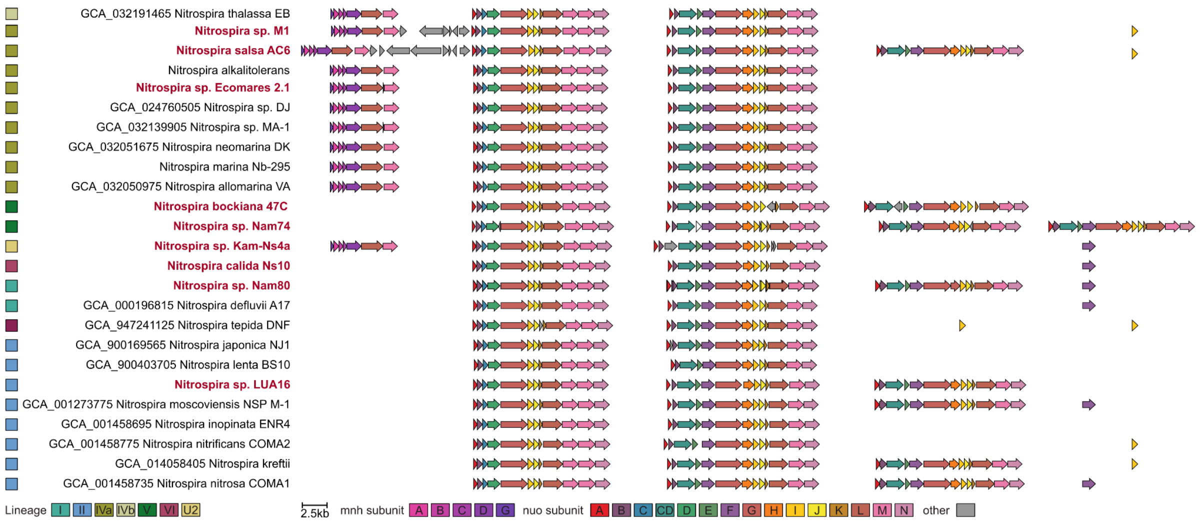

Figure S8. Schematic illustration of gene clusters encoding the NADH-quinone dehydrogenase (*nuo*) and multicomponent Na^+:H^+ antiporter subunits (*mnh*/*mrp*) located next to *nuo* genes in genomes of cultivated *Nitrospirales*. Genes shown closely next to each other are co-localized in the genome and are drawn to scale. The scale bar represents 2.5 kb. Genes for proteins with other functions are shown in grey. The prefix '*Candidatus*' was omitted from the genome names for brevity; for details, see Table S1.

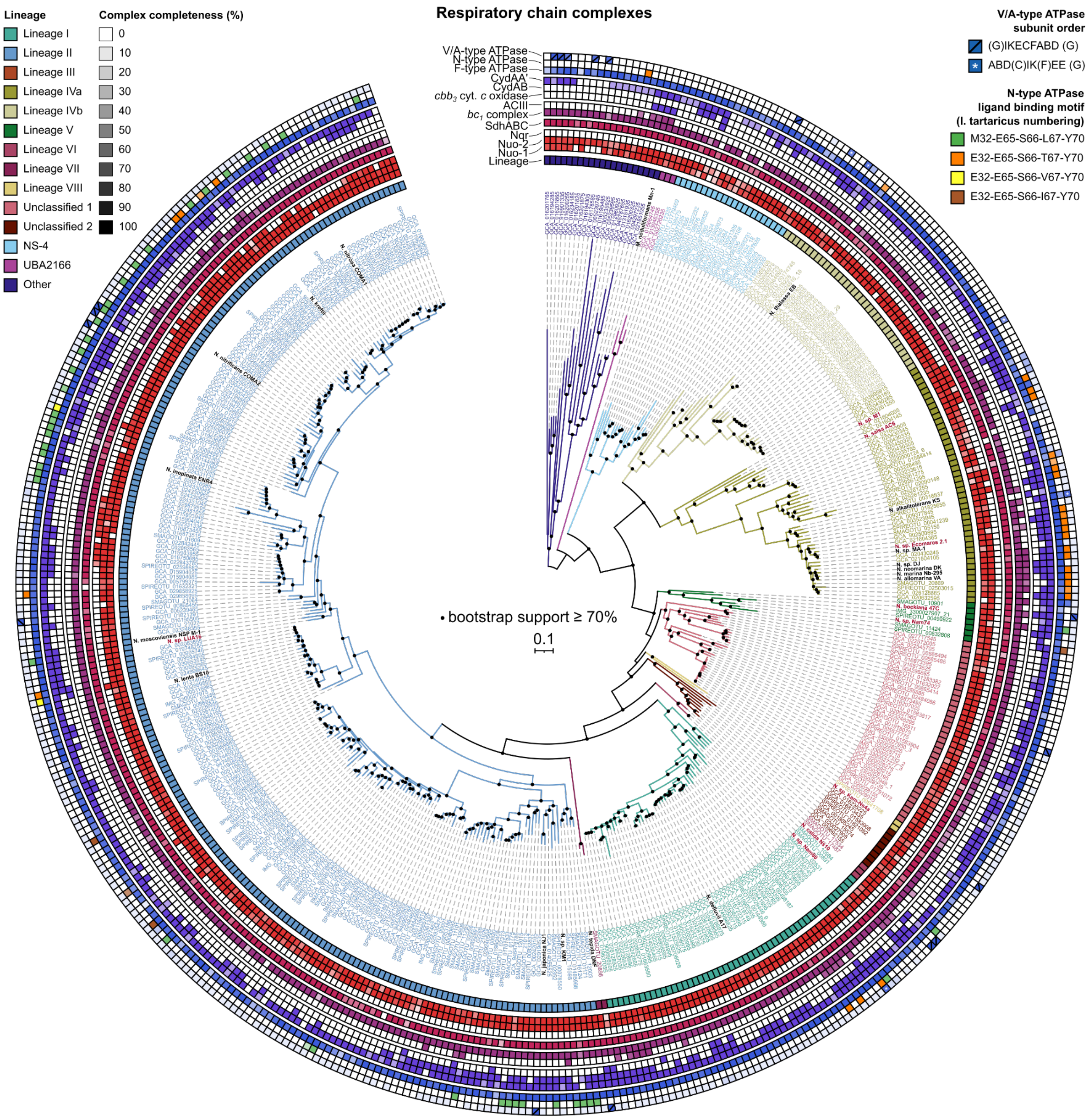

Figure S9. Heatmap showing the presence/absence and completeness of respiratory chain complexes in non-redundant *Nitrospirales* genomes, shown in circles around the same phylogenetic tree as in Figure 1. Completeness of complexes is indicated by the color shade. The subunit order of the genes encoding the V/A-type ATPase is indicated by a diagonal line [(G)IKECFABD (G)] or an asterisk [ABD(C)IK(F)EE (G)] for genomes encoding ≥50% of the genes for the complex. Subunits in parentheses are not present in all genomes, and spaces indicate that the genes are in different locations in the genome. For the N-type ATPases, the square color indicates the ligand binding motif. Black circles indicate bootstrap support ≥70% of 1000 ultrafast bootstrap replicates. The scale bar represents 10% sequence divergence. Tree branches and labels are colored according to the lineage classifications. Newly sequenced genomes are labeled in bold red, genomes of cultivated *Nitrospirales* in

bold black font. The prefix '*Candidatus*' was omitted from the species names for brevity; for details, see Table S1. GTDB representatives belonging to orders other than the *Nitrospirales* within the class *Nitrospiria* are labeled as 'Other'.

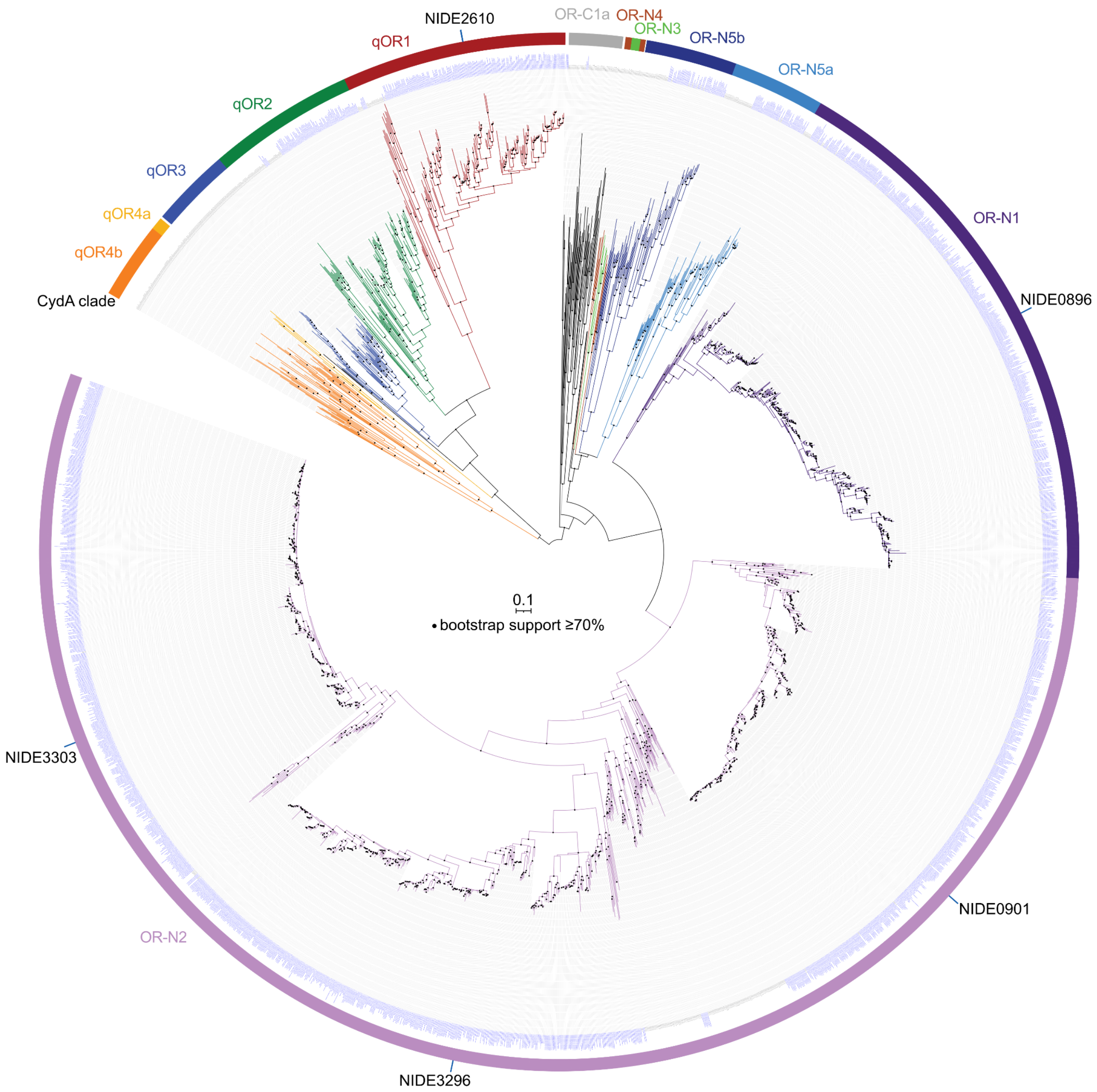

Figure S10. Phylogeny of *bd*-type oxygen reductase CydA sequences. Black circles indicate bootstrap support ≥70%. The phylogenetic tree was calculated using the CydA sequences of the multiple sequence alignment MSA2 by Murali et al. 2021 and CydA clades were labeled accordingly. The tree was rooted using the qOR-type CydA sequences. *Nitrospirales* CydA sequence labels are in blue. The *Nitrospira defluvii* CydA sequences are labeled to indicate the groups that contain the NIDE0901 and NIDE0896 sequences, which are the sequences putatively forming the CydAA' terminal oxidase (Lücker et al., 2010).

```
Ilyobacter tartaricus  (1YCE_1)                                   28 PGVGQGYAA 36   61 QAVAESTGIYSLVI 74
GCA_024330945.1 (JANDJJ010000147.1_3)                             26 PAMAMGRAI 34   59 LAMIESLAIYCLVV 72
GCA_024330965.1 (JANDJL010000027.1_13)                            26 PAVAMGRAI 34   59 LAMLESLAIYCLVV 72
GCA_024331085.1 (JANDJG010000079.1_8)                             26 PAMAMGRAI 34   59 LAMIESLAIYCLVV 72
GCA_024331045.1 (JANDJC010000100.1_3)                             26 PAMAMGRAI 34   59 LAMIESLAIYCLVV 72
GCA_024331125.1 (JANDJI010000001.1_596)                           26 PAVAMGRAI 34   59 LAMIESLAIYCLVV 72
GCA_024331095.1 (JANDJE010000056.1_2)                             26 PAVAMGRAI 34   59 LAMIESLAIYCLVV 72
Nitrospira inopinata ENR4 GCA_001458695.1 (LN885086.1_634)        26 PAVAMGRAI 34   59 LAMIESLAIYCLVV 72
SPIREOTU_00490922 (44277_14)                                      26 PALAMGRAI 34   59 LAMIESLAIYCLVI 72
GCA_946479785.1 (CAMLHQ010000002.1_4)                             26 PALAMGRAI 34   59 LAMIESLAIYCLVI 72
SMAGOTU_01171 (942673_18)                                         26 PAIAMGRAI 34   59 LAMIESLAIYCLVI 72
SMAGOTU_12015 (1384375_4)                                         26 PAMAMGRAI 34   59 LAMIESLAIYCLVI 72
SMAGOTU_16124 (1967723_119)                                       26 PAMAMGRAI 34   59 LAMIESLAIYCLVI 72
SPIREOTU_02374049 (243094_12)                                     26 PAMAMGRVI 34   59 LAMIESLAIYCLVI 72
SMAGOTU_15598 (736282_25)                                         26 PAMAMGRAI 34   59 LAMIESLAIYCLVI 72
SPIREOTU_01485968 (5579473_25)                                    26 PAMAMGRAI 34   59 LAMIESLAIYCLVI 72
SMAGOTU_21029 (771761_5).                                         26 PAMAMGRAI 34   59 LAMIESLAIYCLVI 72
GCA_022843765.1 (JALHNA010000035.1_30)                            26 PAMAMGRAI 34   59 LAMIESLAIYCLVI 72
SPIREOTU_02188117 (36453_89)                                      26 PAMAMGRAI 34   59 LAMIESLAIYCLVI 72
GCA_005116955.1 (SWDI01000001.1_176)                              26 PAMAMGRAI 34   59 LAMIESLAIYCLVI 72
SMAGOTU_21013 (2350769_12)                                        26 PAMAMGRAI 34   59 LAMIESLAIYCLVI 72
GCA_030444405.1 (JARFPM010000019.1_31)                            26 PAMAMGRAI 34   59 LAMIESLAIYCLVI 72
GCA_005116895.1 (SWDO01000004.1_328)                              26 PAMAMGRAI 34   59 LAMIESLAIYCLVI 72
SMAGOTU_02288 (1262850_5)                                         26 PALAMGRAI 34   59 LAMIESLAIYCLVI 72
GCA_019636875.1 (JAHBWR010000004.1_20)                            26 PALAMGRAI 34   59 LAMIESLAIYCLVI 72
Nitrospira sp. LUA16 (LUA.2_1729)                                 26 PAIAMGRAI 34   59 LAMIESLAIYCLVI 72
GCA_029879905.1 (JAOUPN010000001.1_53)                            26 PAIAMGRAI 34   59 LAMIESLAIYCLVI 72
GCA_002451055.1 (DKBD01000220.1_9)                                26 PAIAMGRAI 34   59 LAMIESLAIYCLVI 72
GCA_029879695.1 (JAOUPT010000026.1_9)                             26 PAIAMGRAI 34   59 LAMIESLAIYCLVI 72
SMAGOTU_17534 (345525_24)                                         26 PALAMGRAI 34   59 LAMIESLAIYCLVI 72
GCA_030123565.1 (CP126122.1_359)                                  26 PAIAMGRAI 34   59 LAMIESLAIYCLVV 72
SPIREOTU_00832837 (833859_58)                                     26 PAIAMGRAI 34   59 LAMIESLAIYCLVI 72
GCA_011090425.1 (PHGD01000049.1_9)                                26 PALAMGRAI 34   59 LAMIESLAIYCLVV 72
SMAGOTU_01525 (1298057_8)                                         26 PALAMGWAI 34   59 LAMIESLAIYCLVV 72
SMAGOTU_02352 (706111_2)                                          26 PALAMGRAI 34   59 LAMIESLAIYCLVI 72
GCA_029194675.1 (JARFXP010000014.1_14)                            26 PAIAMGRAI 34   59 LAMIESLAIYCLVI 72
GCA_018971525.1 (JAGXAR010000051.1_19)                            26 PALAMGRAI 34   59 LAMIESLAIYCLVI 72
GCA_018242725.1 (JAFEBI010000001.1_409)                           26 PAIAMGRAI 34   59 LAMIESLAIYCLVI 72
GCA_024281355.1 (JAMXAV010000001.1_49)                            26 PAIAMGRAI 34   59 LAMIESLAIYCLVI 72
GCA_019636915.1 (JAHBWQ010000123.1_8)                             26 PAIAMGRAI 34   59 LAMIESLAIYCLVI 72
SPIREOTU_00841702 (2779730_56).                                   26 PAIAMGRAI 34   59 LAMIESLAIYCLVI 72
SMAGOTU_21038 (919085_9).                                         26 PAIAMGRAI 34   59 LAMIESLAIYCLVI 72
GCA_030123625.1 (CP126121.1_2310)                                 26 PAIAMGRAI 34   59 LAMIESLAIYCLVI 72
GCA_028580895.1 (JAJSDT010000004.1_42)                            26 PAIAMGRAI 34   59 LAMIESLAIYCLVI 72
SPIREOTU_01664340 (137730_29)                                     26 PAIAMGRAI 34   59 LAMIESLAIYCLVI 72
Aphanothece halophytica ApNa (F2Z9N1)                             30 PAIGEGLAL 38   63 MAFVESTAIYCFVI 76
GCA_029261305.1 (JAJDBO010000025.1_6)                             26 PALGEGRSV 34   58 LAMIESTAIYCFVV 71
GCA_029261235.1 (JAJDBS010000226.1_1)                             45 PALGEGRAV 53   78 LAMIESTAIYCFVV 91
GCA_029261255.1 (JAJDBQ010000169.1_5)                             26 PALGEGRAV 34   59 LAMIESTAIYCFVV 72
GCA_945787655.1 (CALZIJ010000015.1_20)                            26 PALGEGRAV 34   59 LAMVESTAIYCFVV 72
GCA_030144965.1 (JAMPUU010000026.1_245)                           26 PALGEGKAV 34   59 LAMVESVAIYCFVV 72
GCA_030692565.1 (JAUYAO010000031.1_38)                            26 PALGEGRAV 34   59 LAMVESVAIYCFVV 72
SPIREOTU_00832808 (470512_3)                                      26 PALGEGRAV 34   59 LAMIESIAIYCFVV 72
SPIREOTU_01600253 (954186_51)                                     26 PALGEGRAV 34   59 LAMIESIAIYCFVV 72
SPIREOTU_01126182 (772433_2)                                      26 PALGEGRAV 34   59 LAMIESIAIYCFVV 72
SPIREOTU_01948526 (172290_72)                                     26 PALGEGRAV 34   59 LAMIESIAIYCFVV 72
Nitrospira marina Nb-295 (N.marina_2833)                          26 PALGEGRAT 34   59 LAMIESIAIYAFVV 72
Nitrospira allomarina VA GCA_032050975.1 (CP116967.1_1970)        26 PALGEGRAT 34   59 LAMIESTAIYAFVV 72
Nitrospira sp. DJ  GCA_024760505.1 (CP058931.1_2059)              26 PALGEGKAV 34   59 LAMIESTAIYCFVV 72
SPIREOTU_00316837 (1375136_13).                                   26 PALGEGRAV 34   59 LAMIESTAIYCFVV 72
SMAGOTU_05155 (285842_16)                                         26 PALGEGRAV 34   59 LAMIESTAIYCFVV 72
Nitrospira sp. Ecomares 2.1 (NITECO.1_2913)                       26 PALGEGRAI 34   59 LAMIESTAIYCFVV 72
GCA_021604365.1 (BQIZ01000198.1_3)                                26 PALGEGRAI 34   59 LAMIESTAIYCFVV 72
GCA_902500695.1 (CABVRQ010000007.1_76)                            26 PALGEGRAV 34   59 LAMIESTAIYCFVV 72
Nitrospira sp. MA-1 GCA_032139905.1 (JAQJDB010000007.1_592)       26 PALGEGRAV 34   59 LAMIESTAIYCFVV 72
GCA_021604105.1 (BQIW01000066.1_29)                               26 PALGEGKAI 34   59 LAMIESTAIYCFVV 72
GCA_020430345.1 (JAGQKA010000002.1_49)                            26 PALGEGKAV 34   59 LAMIESTAIYCFVV 72
GCA_020430325.1 (JAGQKE010000028.1_17)                            26 PALGEGKAV 34   59 LAMIESTAIYCFVV 72
GCA_020430305.1 (JAGQKD010000178.1_14)                            26 PALGEGKAV 34   59 LAMIESTAIYCFVV 72
GCA_026128885.1 (JAHCJD010000005.1_23)                            26 PALGEGRAV 34   59 LAMIESTAIYCFVI 72
SPIREOTU_02503015 (241727_1)                                      26 PALGEGRAI 34   59 LAMIESTAIYCFVV 72
Nitrospira alkalitolerans KS (1714900079_2)                       48 PALGEGRAV 56   81 LAMIESTAIYCFVV 94
SMAGOTU_20869 (505897_9)                                          26 PALGEGRAV 34   59 LAMIESTAIYCFVV 72
GCA_029858925.1 (JAOUEB010000059.1_7)                             26 PALGEGRAV 34   59 LAMIESTAIYCFVV 72
GCA_029858925.1 (JAOUEB010000463.1_3)                             26 PALGEGRAV 34   59 LAMIESTAIYCFVV 72
GCA_029865225.1 (JAOUHJ010000034.1_12)                            26 PALGEGKAV 34   59 LAMIESTAIYCFVV 72
GCA_029881435.1 (JAOUMR010000049.1_25)                            26 PALGEGKAV 34   59 LAMIESTAIYCFVV 72
GCA_016715825.1 (JADJXO010000007.1_119)                           26 PALGEGRAV 34   59 LAMIESTAIYCFVV 72
GCA_017879665.1 (JAAEYB010000008.1_34)                            26 PALGEGRAV 34   59 LAMIESTAIYCFVV 72
Nitrospira kreftii GCA_014058405.1 (CP047423.1_3012)              26 PALGEGRAV 34   59 LAMIESTAIYCFVV 72
SPIREOTU_00498187 (465236_52)                                     26 PALAEGRAV 34   59 LAMIESTAIYCFVV 72
GCA_902500785.1 (CABVRW010000026.1_45).                           26 PALAEGRAV 34   59 LAMIESTAIYCFVV 72
GCA_030653545.1 (JAURZE010000005.1_167)                           26 PALGEGKAV 34   59 LAMIESTAIYCFVV 72
                                                                     *.:. *            *. ** .**.:*:
```

Figure S11. CLUSTAL O (v1.2.4) multiple sequence alignment of *Nitrospirales* AtpE sequences with Na^+ binding residues highlighted according to *Ilyobacter tartaricus* numbering: Gln32, Glu65, Ser66, Thr67, and Tyr70 [35]. The *Ilyobacter tartaricus* Q EST--Y motif is highlighted in red. Amino acids that differ from this motif are highlighted in yellow. The prefix '*Candidatus*' was omitted from the species names for brevity; for details, see Table S1.

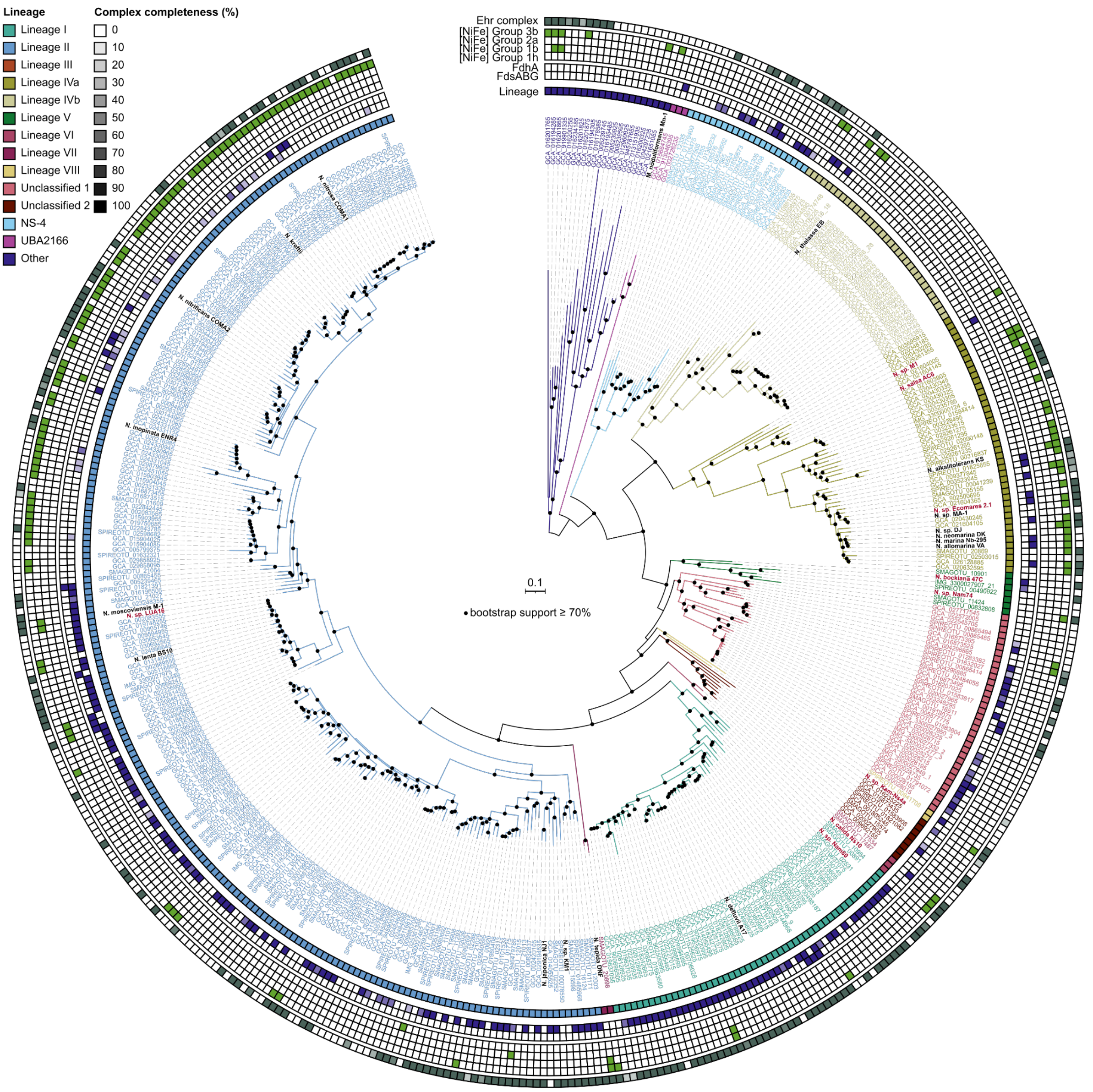

Figure S12. Heatmap showing the distribution and completeness of the formate dehydrogenases, hydrogenases, and Ehr complex in non-redundant *Nitrospirales* genomes, shown in circles around the same phylogenetic tree as in Figure 1. Completeness of complexes is indicated by the color shade. Black circles indicate bootstrap support ≥70% of 1000 ultrafast bootstrap replicates. The scale bar represents 10% sequence divergence. Tree branches and labels are colored according to the lineage classifications. Newly sequenced genomes are labeled in bold red, genomes of cultivated *Nitrospirales* in bold black font. The prefix '*Candidatus*' was omitted from the species names for brevity; for details, see Table S1. Abbreviations: FDH = canonical formate dehydrogenase; FdhA = *Nitrospira marina*-like putative formate dehydrogenase; [NiFe] Group 1b, 1h, 2a, and 3b = [NiFe] hydrogenases belonging to the groups 1b, 1h, 2a, and 3b; Ehr complex = Ech hydrogenase-related complex.

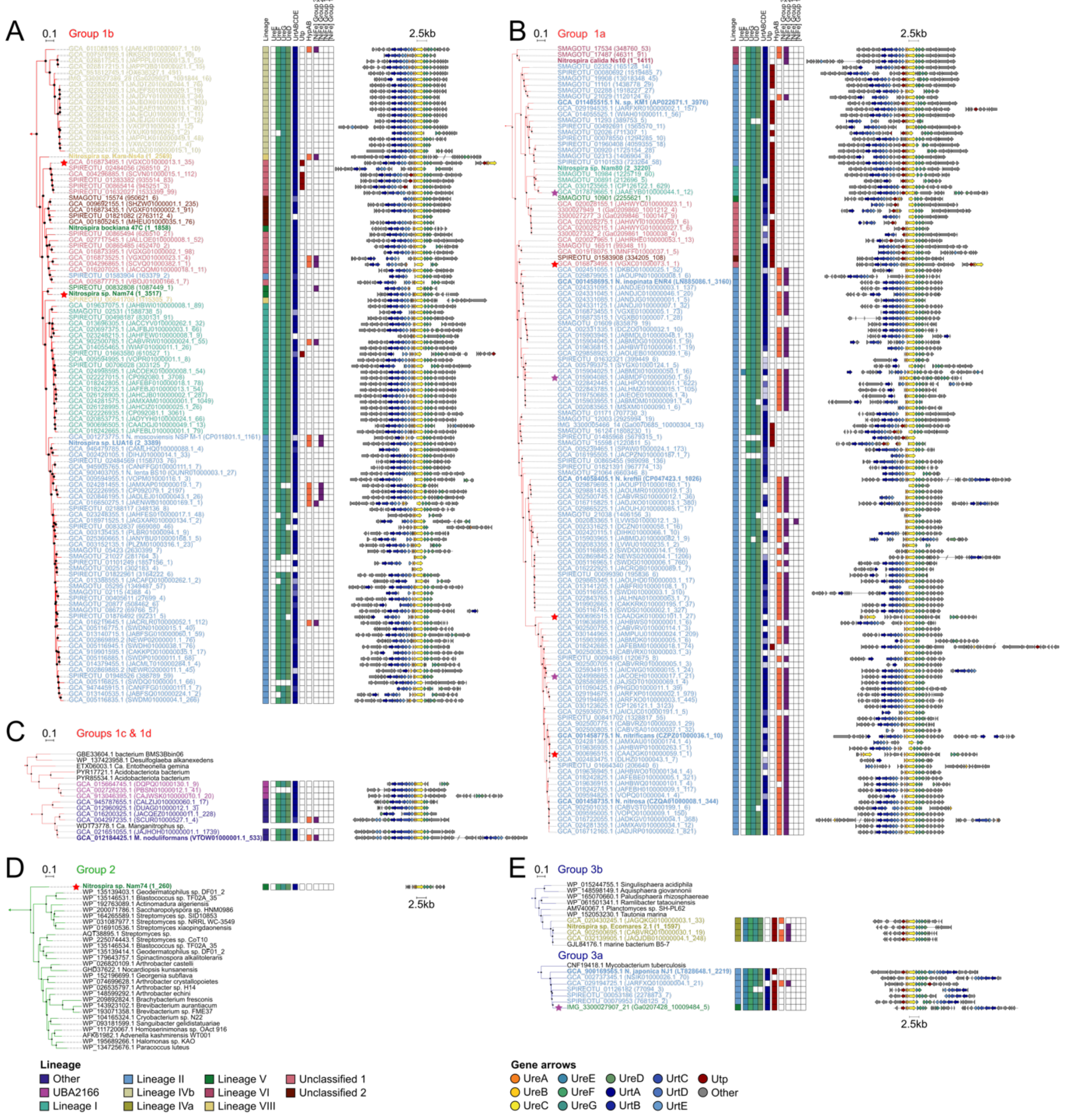

Figure S13. Partial UreC trees with additional information on the lineage, distribution of urease accessory proteins (green), urea transporters (blue and red), hydrogenase accessory proteins (orange), and [NiFe] hydrogenases groups (purple), as well as a schematic illustration of the genomic locations encoding the urease-related genes in each genome. Genes are drawn to scale, with the respective scale bars representing 2.5 kb. The phylogenetic trees are the same as in Figure 4C. Sequence labels are colored according to the lineage classifications, with the exception of the newly sequenced genomes and genomes of cultivated *Nitrospirales*, which are shown in bold font. Tree lines are colored according to the three groups shown in Figure 4A. Black circles indicate bootstrap support ≥70% of 1000

ultrafast bootstrap replicates. The tree scale bars represent 10% sequence divergence. Red stars mark sequences of genomes that have multiple complete UreC copies. Purple stars mark genomes in which a second, partial *ureC* gene was found. **A)** Group 1b UreC sequences. **B)** Group 1a UreC sequences. **C)** Group 1c and 1d UreC sequences. **D)** Group 2 UreC sequences. **E)** Group 3 UreC sequences. Abbreviations: UreFGDE = urease accessory proteins; UrtABCDE = ATP-dependent ABC-type urea transporter; Utp = urea transporter (UT) family protein.

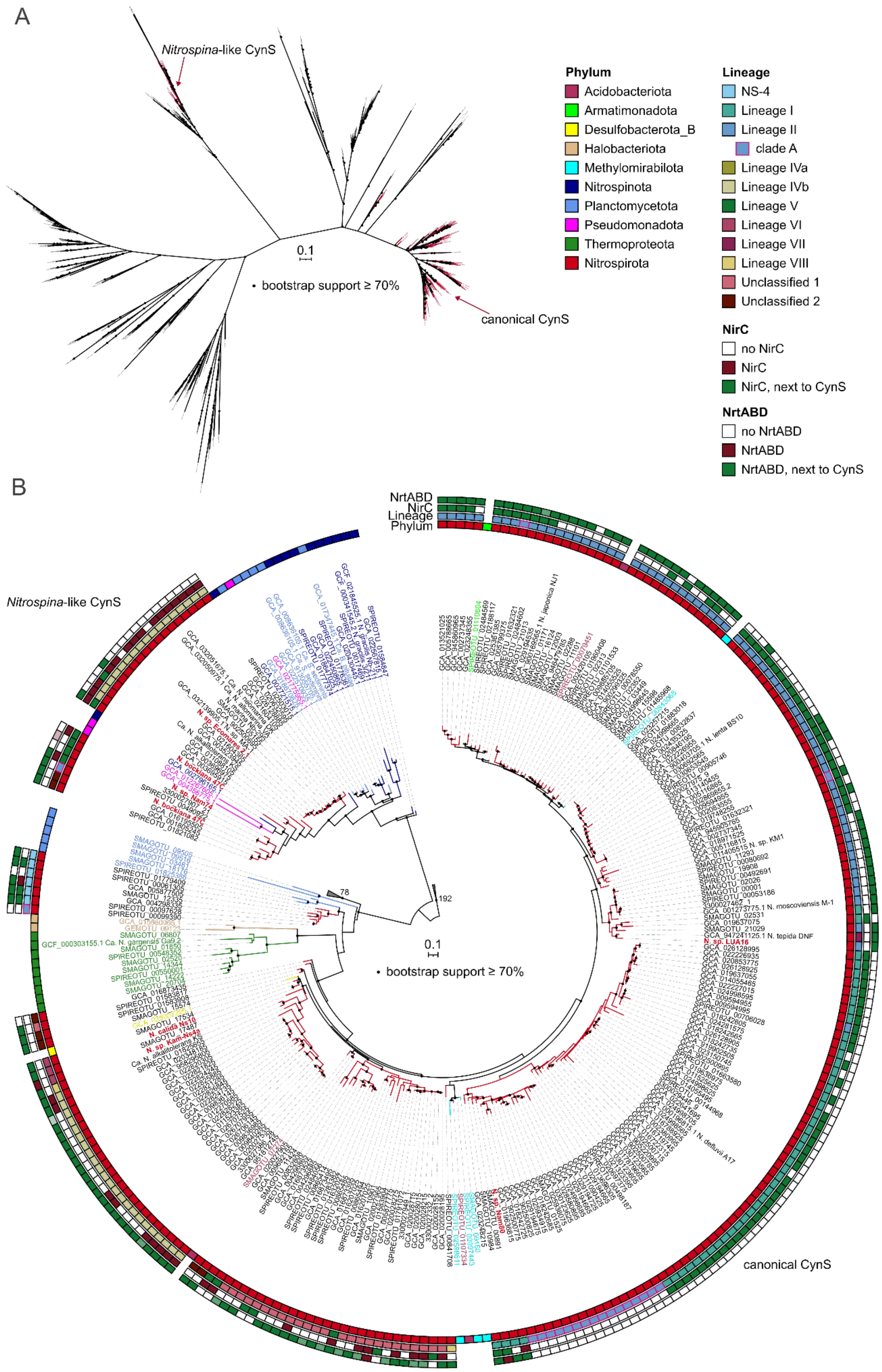
A
Nitrospina-like CynS
0.1
• bootstrap support ≥ 70%
canonical CynS
Phylum
Acidobacteriota
Armatimonadota
Desulfobacterota_B
Halobacteriota
Methylomirabilota
Nitrospinota
Planctomycetota
Pseudomonadota
Thermoproteota
Nitrospirota
Lineage
NS-4
Lineage I
Lineage II
clade A
Lineage IVa
Lineage IVb
Lineage V
Lineage VI
Lineage VII
Lineage VIII
Unclassified 1
Unclassified 2
NirC
no NirC
NirC
NirC, next to CynS
NrtABD
no NrtABD
NrtABD
NrtABD, next to CynS
B
NrtABD
NirC
Lineage
Phylum
Nitrospina-like CynS
78
192
0.1
• bootstrap support ≥ 70%
canonical CynS

Figure S14. Phylogeny of cyanases in *Nitrospirales* genomes. **A)** Unrooted CynS tree with the *Nitrospirales* sequences indicated by red branches. **B)** Circular representation of the midpoint-rooted CynS tree. Sequence labels and tree lines of reference sequences are colored according to the GTDB-Tk phylum classifications. The newly sequenced genomes are shown in bold red font. The circles around the tree from the innermost to the outermost ring show the phylum (GTDB-Tk classification), *Nitrospirales* lineage, and presence/absence of NirC and NrtABD sequences. For the latter, the color intensity indicates the number of subunits present. Black circles in both trees represent bootstrap support ≥70% of 1000 ultrafast bootstrap replicates, the scale bars 10% sequence divergence.

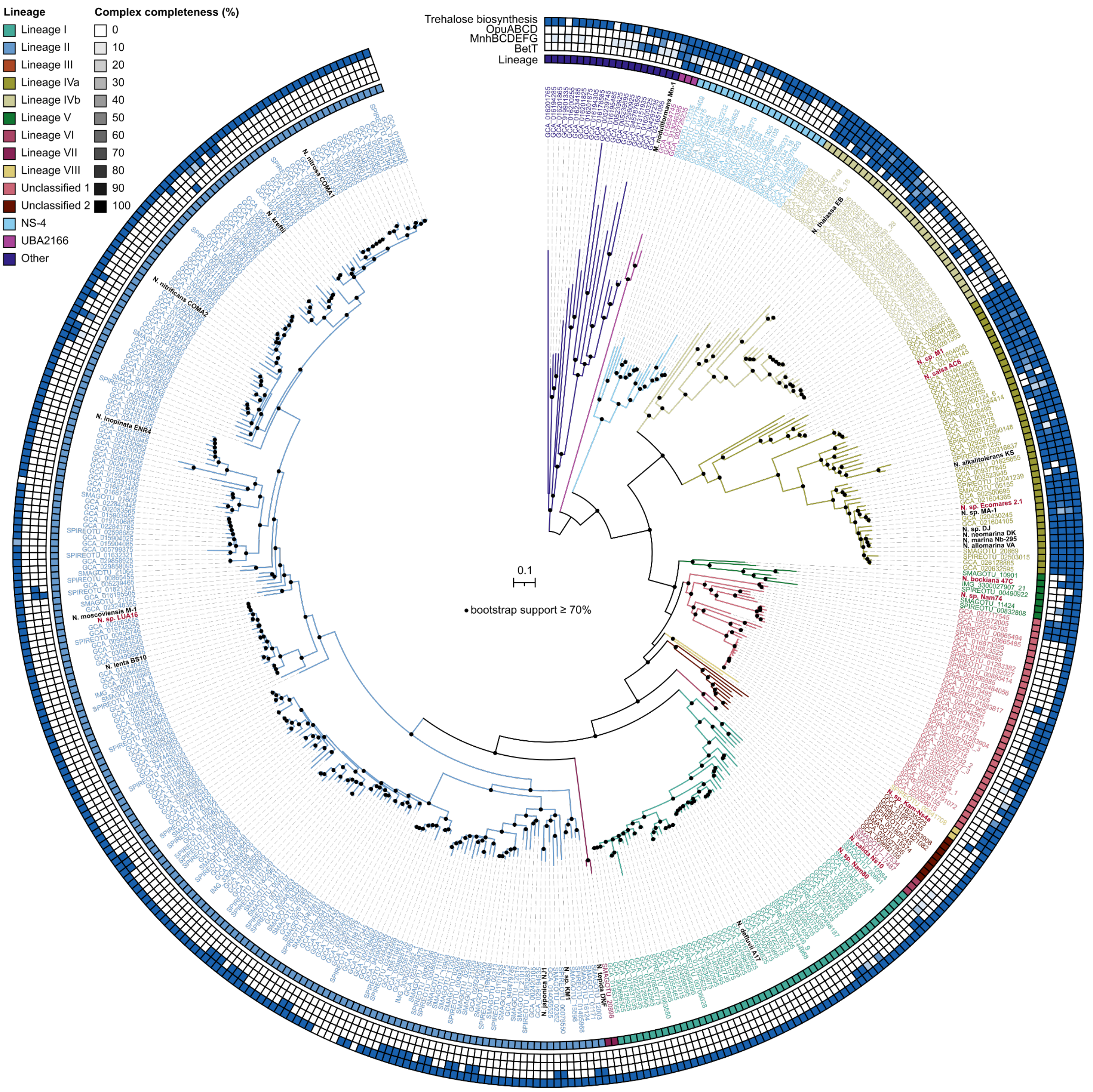

Figure S15. Heatmap showing the distribution and completeness of genes involved in osmoprotection in non-redundant *Nitrospirales* genomes, shown in circles around the same phylogenetic tree as in Figure 1. Completeness of complexes is indicated by the color shade. Black circles indicate bootstrap support ≥70% of 1000 ultrafast bootstrap replicates. The scale bar represents 10% sequence divergence. Tree branches and labels are colored according to the lineage classifications. Newly sequenced genomes are labeled in bold red, genomes of cultivated *Nitrospirales* in bold black font. The prefix '*Candidatus*' was omitted from the species names for brevity; for details, see Table S1. Abbreviations: BetT = choline/glycine/proline betaine transport protein; MnhBCDEFG = multicomponent Na^+:H^+ antiporter; OpuABCD = osmoprotectant transport system.

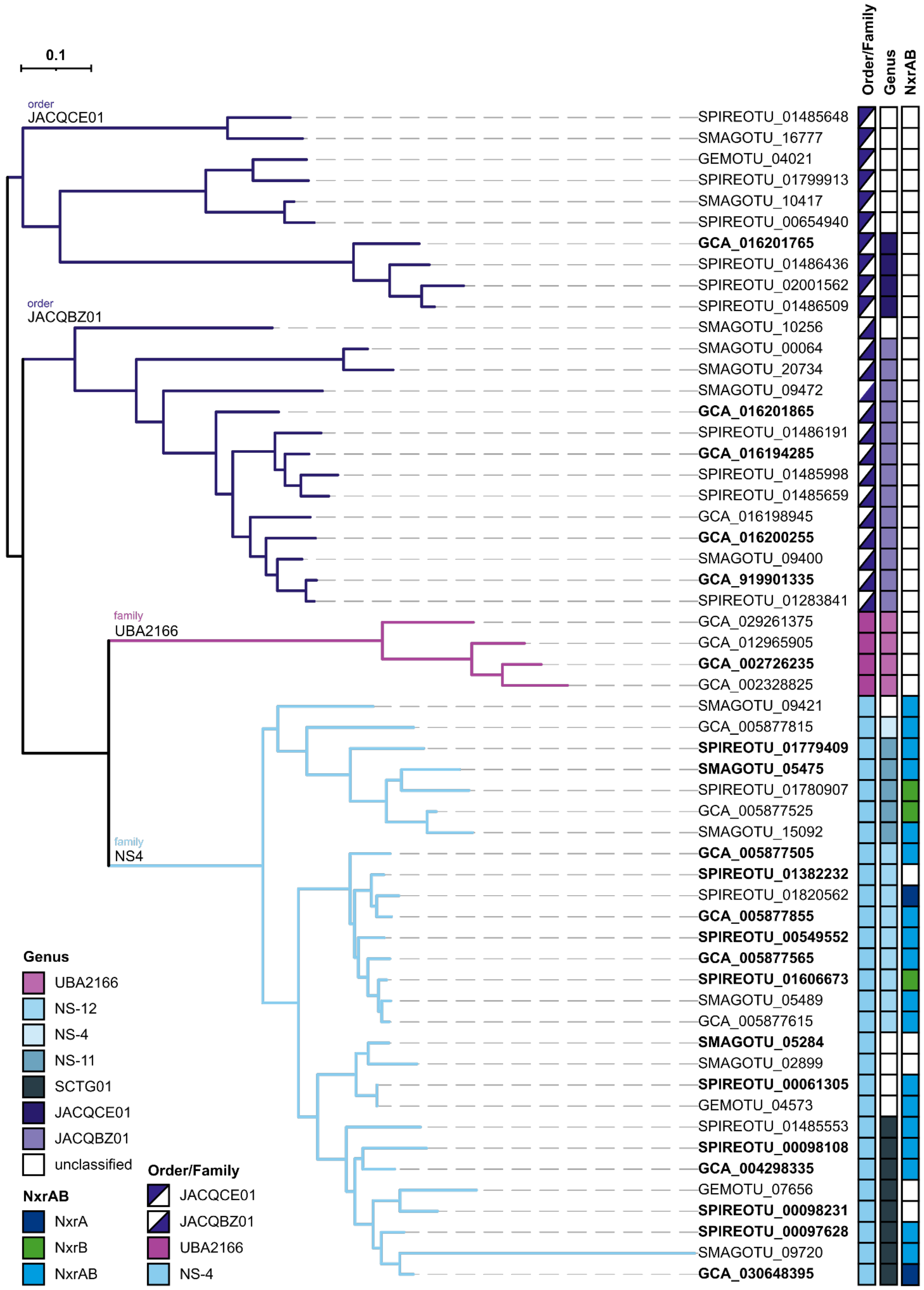

Figure S16. Phylogenetic tree based on 71 bacterial marker genes, with emphasis on genomes around the acquisition of NXR in the *Nitrospirota* phylum. The scale bar represents 10% sequence divergence. The genomes are members of the JACQCE01 (n=10) and JACQBZ01 (n=14) orders, and UBA2166 (n=4) and NS-4 (n=28) families. Genomes that are also part of the main dataset (Table S4) are marked in bold.

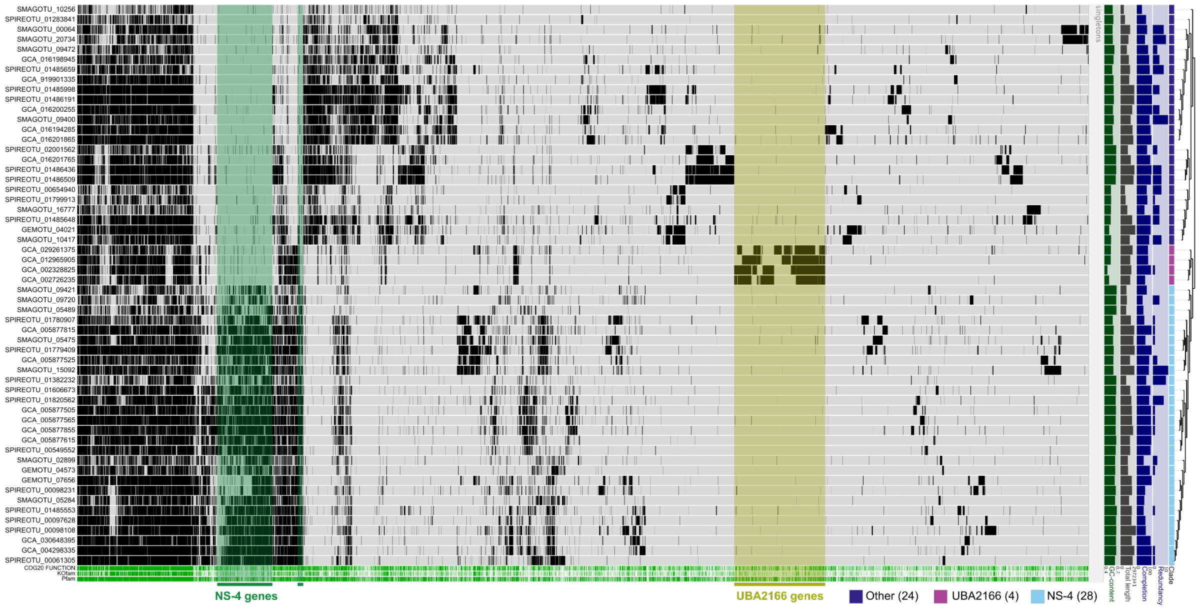

Figure S17. Heatmap indicating the presence (black) and absence (gray) of homologous gene clusters (columns) across 56 genomes (rows) of the JACQCE01 and JACQBZ01 orders and UBA2166 and NS-4 families. Columns representing genes present in a single genome were omitted. The columns in the heatmap are ordered by the frequency of their occurrence in the genomes. The rows are ordered by the frequencies of their gene cluster content. Columns highlighted in green were identified as enriched in the NS-4 genomes, whereas columns highlighted in yellow are enriched in the UBA2166 genomes. The gene clusters represented by these columns were extracted and filtered to remove any clusters containing genes encoded by JACQCE01 and JACQBZ01 genomes, resulting in 343 NS-4 specific (Table S9) and 544 UBA2166 specific clusters (Table S7).

Supplementary Table Legends

Table S1. Overview of cultivated *Nitrospirales* genome sequences, their accession number (if available), number of contigs, lineage (if applicable), and isolation/enrichment source. In addition, the reference and corresponding DOI are listed. Genomes newly sequenced in this study are highlighted in green.

Table S2. Information on the NanoPore sequencing of the *Nitrospirales* genomes including the amount of input DNA, the ligation and barcoding kits, as well as the basecalling software used.

Table S3. Overview of all genomes used in this study to select non-redundant genomes (≥75% estimated completeness, ≤10% estimated redundancy), together with GTDB-Tk, CheckM, and dRep results. Genomes newly sequenced in this study are highlighted in green. Selected non-redundant genome accessions are written in green font and sorted according to the phylogenomic tree shown in Figure 1 by the "index" column. Redundant genome accession numbers are written in blue font. Genomes that were excluded from further analyses due to their taxonomic classification or estimated completeness and redundancy are marked in red and orange font, respectively.

Table S4. Information on the lineage, habitat, accession number, and publication for each of the non-redundant *Nitrospirales* genomes (≥75% estimated completeness, ≤10% estimated redundancy). The index column is in the order according to the phylogenomic tree shown in Figure 1. Newly sequenced genomes are highlighted in green.

Table S5. DRAM annotation results. Gene calling was performed with prodigal [36] and annotation against the KOfam [37] and Pfam [38] databases.

Table S6. Manually curated annotation results of key genes, using DRAM annotations and blastp (2.13.0+) [39] searches of representative proteins (e-value ≤0.00001, bitscore ≥30, percent identity ≥30%, query cover ≥80%, with manual curation of results). Newly sequenced genomes are highlighted in green.

Table S7. 16S rRNA gene sequence identities (%) within each *Nitrospira* lineage for sequences >1200 bp.

Table S8. Average amino acid identities (AAI; %) between genomes within each *Nitrospira* lineage.

Table S9. Clusters of homologous genes specific to UBA2166 family genomes, and absent in the genomes of the JACQCE01 and JACQBZ01 order, and NS-4 family genomes, with consensus KEGG, COG, and PFAM annotations.

Table S10. Detection of the UBA2166 family across 248,905 metagenomes included in the sandpiper database (version 0.3.0).

Table S11. Clusters of homologous genes specific to NS-4 family genomes, and absent in the genomes of the JACQCE01 and JACQBZ01 order, and the UBA2166 family genomes, with consensus KEGG, COG, and PFAM annotations.

Table S12. Clusters of homologous genes specific to NS-4 family genomes, and conserved in over 300 of the NXR-containing *Nitrospirales* genomes, with consensus KEGG, COG, and PFAM annotations and the respective gene identifiers and annotations in *N. defluvii* and *N. moscoviensis*.

References

1. Renshaw MA, Olds BP, Jerde CL, et al. The room temperature preservation of filtered environmental DNA samples and assimilation into a phenol–chloroform–isoamyl alcohol DNA extraction. *Mol Ecol Resour* 2015; **15**: 168–176.
2. Spieck E, Spohn M, Wendt K, et al. Extremophilic nitrite-oxidizing *Chloroflexi* from Yellowstone hot springs. *ISME J* 2020; **14**: 364–379.
3. Keuter S, Koch H, Sass K, et al. Some like it cold: the cellular organization and physiological limits of cold-tolerant nitrite-oxidizing *Nitrotoga*. *Environ Microbiol* 2022; **4**: 2059-2077.
4. Poghosyan L, Koch H, Frank J, et al. Metagenomic profiling of ammonia- and methane-oxidizing microorganisms in two sequential rapid sand filters. *Water Res* 2020; **185**: 116288.
5. Wick RR, Judd LM, Gorrie CL, et al. Unicycler: Resolving bacterial genome assemblies from short and long sequencing reads. *PLOS Comput Biol* 2017; **13**: e1005595.
6. Eren AM, Esen ÖC, Quince C, et al. Anvi'o: an advanced analysis and visualization platform for 'omics data. *PeerJ* 2015; **3**: e1319.
7. Seemann T. Prokka: Rapid prokaryotic genome annotation. *Bioinformatics* 2014; **30**: 2068–2069.
8. Koren S, Walenz BP, Berlin K, et al. Canu: scalable and accurate long-read assembly via adaptive k-mer weighting and repeat separation. *Genome Res* 2017; **27**: 722–736.
9. Li H. Minimap2: Pairwise alignment for nucleotide sequences. *Bioinformatics* 2018; **34**: 3094–3100.
10. Vaser R, Sović I, Nagarajan N, et al. Fast and accurate de novo genome assembly from long uncorrected reads. *Genome Res* 2017; **27**: 737–746.
11. Edgar RC. Search and clustering orders of magnitude faster than BLAST. *Bioinformatics* 2010; **26**: 2460–2461.
12. Edgar RC. MUSCLE: Multiple sequence alignment with high accuracy and high throughput. *Nucleic Acids Res* 2004; **32**: 1792–1797.
13. Ludwig W. ARB: a software environment for sequence data. *Nucleic Acids Res* 2004; **32**: 1363–1371.
14. Jones P, Binns D, Chang H-Y, et al. InterProScan 5: genome-scale protein function classification. *Bioinformatics* 2014; **30**: 1236–1240.

15. Capella-Gutiérrez S, Silla-Martínez JM, Gabaldón T. trimAl: A tool for automated alignment trimming in large-scale phylogenetic analyses. *Bioinformatics* 2009; **25**: 1972–1973.
16. Kalyaanamoorthy S, Minh BQ, Wong TKF, et al. ModelFinder: Fast model selection for accurate phylogenetic estimates. *Nat Methods* 2017; **14**: 587–589.
17. Nguyen LT, Schmidt HA, Von Haeseler A, et al. IQ-TREE: A fast and effective stochastic algorithm for estimating maximum-likelihood phylogenies. *Mol Biol Evol* 2015; **32**: 268–274.
18. Mao X, Chen J, van Oosterhout C, et al. Diversity, prevalence, and expression of cyanase genes (*cynS*) in planktonic marine microorganisms. *ISME J* 2021; 6–9.
19. Rasko DA, Myers GSA, Ravel J. Visualization of comparative genomic analyses by BLAST score ratio. *BMC Bioinformatics* 2005; **6**: 2.
20. Speth DR, Orphan VJ. Metabolic marker gene mining provides insight in global *mcrA* diversity and, coupled with targeted genome reconstruction, sheds further light on metabolic potential of the *Methanomassiliicoccales*. *PeerJ* 2018; **6**: e5614.
21. Price MN, Dehal PS, Arkin AP. FastTree 2 - Approximately maximum-likelihood trees for large alignments. *PLoS One* 2010; **5**: e9490.
22. Minh BQ, Schmidt HA, Chernomor O, et al. IQ-TREE 2: New models and efficient methods for phylogenetic inference in the genomic era. *Mol Biol Evol* 2020; **37**: 1530–1534.
23. Hoang DT, Chernomor O, von Haeseler A, et al. UFBoot2: Improving the Ultrafast Bootstrap Approximation. *Mol Biol Evol* 2018; **35**: 518–522.
24. Edgar RC. Muscle5: High-accuracy alignment ensembles enable unbiased assessments of sequence homology and phylogeny. *Nat Commun* 2022; **13**: 6968.
25. Murali R, Gennis RB, Hemp J. Evolution of the cytochrome *bd* oxygen reductase superfamily and the function of CydAA' in Archaea. *ISME J* 2021; **15**: 3534–3548.
26. Parks DH, Imelfort M, Skennerton CT, et al. CheckM: assessing the quality of microbial genomes recovered from isolates, single cells, and metagenomes. *Genome Res* 2015; **25**: 1043–1055.
27. Lee MH, Mulrooney SB, Renner MJ, et al. *Klebsiella aerogenes* urease gene cluster: sequence of *ureD* and demonstration that four accessory genes (*ureD*, *ureE*, *ureF*, and *ureG*) are involved in nickel metallocenter biosynthesis. *J Bacteriol* 1992; **174**: 4324–4330.
28. Voland P, Weeks DL, Marcus EA, et al. Interactions among the seven *Helicobacter*

pylori proteins encoded by the urease gene cluster. *Am J Physiol Liver Physiol* 2003; **284**: G96–G106.

29. Mulrooney SB, Ward SK, Hausinger RP. Purification and properties of the *Klebsiella aerogenes* UreE metal-binding domain, a functional metallochaperone of urease. *J Bacteriol* 2005; **187**: 3581–3585.
30. Benoit SL, Mehta N, Weinberg MV, et al. Interaction between the *Helicobacter pylori* accessory proteins HypA and UreE is needed for urease maturation. *Microbiology* 2007; **153**: 1474–1482.
31. Koch H, Lücker S, Albertsen M, et al. Expanded metabolic versatility of ubiquitous nitrite-oxidizing bacteria from the genus *Nitrospira*. *Proc Natl Acad Sci* 2015; **112**: 11371–11376.
32. Zambelli B, Musiani F, Benini S, et al. Chemistry of Ni^{2+} in urease: Sensing, trafficking, and catalysis. *Acc Chem Res* 2011; **44**: 520–530.
33. Witte CP, Isidore E, Tiller SA, et al. Functional characterisation of urease accessory protein G (ureG) from potato. *Plant Mol Biol* 2001; **45**: 169–179.
34. Brayman TG, Hausinger RP. Purification, characterization, and functional analysis of a truncated *Klebsiella aerogenes* UreE urease accessory protein lacking the histidine-rich carboxyl terminus. *J Bacteriol* 1996; **178**: 5410–5416.
35. Meier T, Krah A, Bond PJ, et al. Complete ion-coordination structure in the rotor ring of Na^+-dependent F-ATP synthases. *J Mol Biol* 2009; **391**: 498–507.
36. Hyatt D, Chen G-L, LoCascio PF, et al. Prodigal: prokaryotic gene recognition and translation initiation site identification. *BMC Bioinformatics* 2010; **11**: 119.
37. Aramaki T, Blanc-Mathieu R, Endo H, et al. KofamKOALA: KEGG Ortholog assignment based on profile HMM and adaptive score threshold. *Bioinformatics* 2020; **36**: 2251–2252.
38. El-Gebali S, Mistry J, Bateman A, et al. The Pfam protein families database in 2019. *Nucleic Acids Res* 2019; **47**: D427–D432.
39. Altschul SF, Gish W, Miller W, et al. Basic local alignment search tool. *J Mol Biol* 1990; **215**: 403–410.
